# Different neural circuitry is involved in physiological and psychological stress-induced PTSD-like “nightmares” in rats

**DOI:** 10.1038/srep15976

**Published:** 2015-11-04

**Authors:** Bin Yu, Su-Ying Cui, Xue-Qiong Zhang, Xiang-Yu Cui, Sheng-Jie Li, Zhao-Fu Sheng, Qing Cao, Yuan-Li Huang, Ya-Ping Xu, Zhi-Ge Lin, Guang Yang, Jin-Zhi Song, Hui Ding, Yong-He Zhang

**Affiliations:** 1 Department of pharmacology, Peking University, School of Basic Medical Science, 38 Xueyuan Road, Beijing, 100191, China

## Abstract

Posttraumatic nightmares are a core component of posttraumatic stress disorder (PTSD) and mechanistically linked to the development and maintenance of this disorder, but little is known about their mechanism. We utilized a communication box to establish an animal model of physiological stress (foot-shock [FS]) and psychological stress (PS) to mimic the direct suffering and witnessing of traumatic events. Twenty-one days after traumatic stress, some of the experimental animals presented startled awakening (i.e., were startled awake by a supposed “nightmare”) with different electroencephalographic spectra features. Our neuroanatomical results showed that the secondary somatosensory cortex and primary auditory cortex may play an important role in remote traumatic memory retrieval in FS “nightmare” (FSN) rats, whereas the temporal association cortex may play an important role in PS “nightmare” (PSN) rats. The FSN and PSN groups possessed common emotion evocation circuits, including activation of the amygdala and inactivation of the infralimbic prefrontal cortex and ventral anterior cingulate cortex. The decreased activity of the granular and dysgranular insular cortex was only observed in PSN rats. The present results imply that different types of stress may cause PTSD-like “nightmares” in rodents and identified the possible neurocircuitry of memory retrieval and emotion evocation.

The *Diagnostic and Statistical Manual of Mental Disorders*, 5th edition, specifically delineates “traumatic nightmares” among intrusion symptoms of posttraumatic stress disorder (PTSD)^1^. In contrast to normal dreams, posttraumatic nightmares often incorporate actual memories of frightening experiences. The recurring traumatic nightmares appear to reinforce the memory of trauma and contribute to the dreamer’s distress. Although temporal relationships between trauma exposure, PTSD, and nightmares are complex, emerging evidence supports the notion of nightmares as a core component of PTSD^2^ that is mechanistically linked to its development and maintenance^3^. A growing body of research has linked the prevalence and frequency of posttraumatic nightmares and distress associated with it to a broad spectrum of mental health pathologies^4^. However, the pathogenesis of PTSD-like nightmares remains unclear.

PTSD can be triggered not only in people who personally experienced traumatic events but also in those who witness them^5^,^6^. In the present study, we utilized a communication box to establish an animal model that mimics physiological stress and psychological stress in individuals with PTSD. This model simulates people who experience or witness a traumatic event. Psychological stress (PS) rats were exposed to visual, olfactory, and auditory stimuli (e.g., struggling, vocalizing, defecating, urinating, and jumping) that were emitted by physiological stress (foot-shock [FS]) rats^7^. Considering epidemiological data indicate that women are more likely than men to develop PTSD or posttraumatic nightmares following trauma exposure^4^,^8^, we used female Sprague-Dawley rats in proestrus in the animal model (Table S1). Some of the rats that were exposed to FS or PS 21 days previously presented startled awakening from sleep (Video S1, S2, and S3). This behavioral phenomenon of being startled awake involved the rats’ suddenly waking up from sleep with jumping behavior under undisturbed conditions. We speculated that “startled awakening” behavior may mimic the phenomenon of posttraumatic nightmares.

Dreaming represents the conscious experience of brain mechanisms that perform offline emotional and memory reprocessing during sleep^9^. Depending on whether the memories are spatial or emotional, recent and remote memories differentially activate the hippocampus and neocortex^10^,^11^. As memories age, neocortical areas become more engaged, and specific neocortical regions come online and mediate access to remote memory^10^,^11^,^12^,^13^. Based on the sensory stimuli that rats received during the modeling process, we speculated that the neocortical subregions that are recruited in remote fear memory include the somatosensory, auditory, visual, and rhinal cortices and association cortex. Additionally, compared with other dreams, dreams after experiencing trauma elicit more generally negative emotions, including anxiety, threat, and aggression, and these appear to be most pronounced in PTSD^14^. We assessed c-Fos expression in fear-related neurocircuitry, including the amygdala, prefrontal cortex (PFC), anterior cingulate cortex (ACC), insular cortex, lateral habenula (LHb), and paraventricular thalamic nucleus (PVT), to explore whether these areas are also involved in regulating the phenomenon of being startled awake.

In the present study, we analyzed EEG spectra just before the rats were startled awake to ascertain in which state of the cortical electrical activity the FS and PS-induced startled awakening were occurrence. We also examined whether the remote traumatic memories were retrieved or the negative emotions were evoked by mapping the c-Fos protein in possible memory storage and emotional evocation related brain regions. The aims were to investigate the mechanisms underlying the FS and PS-induced startled awakening and whether there were differences.

## Results

### Physiological and psychological stress caused some rats to present startled awakening with different EEG spectra features

**Figure 1:**
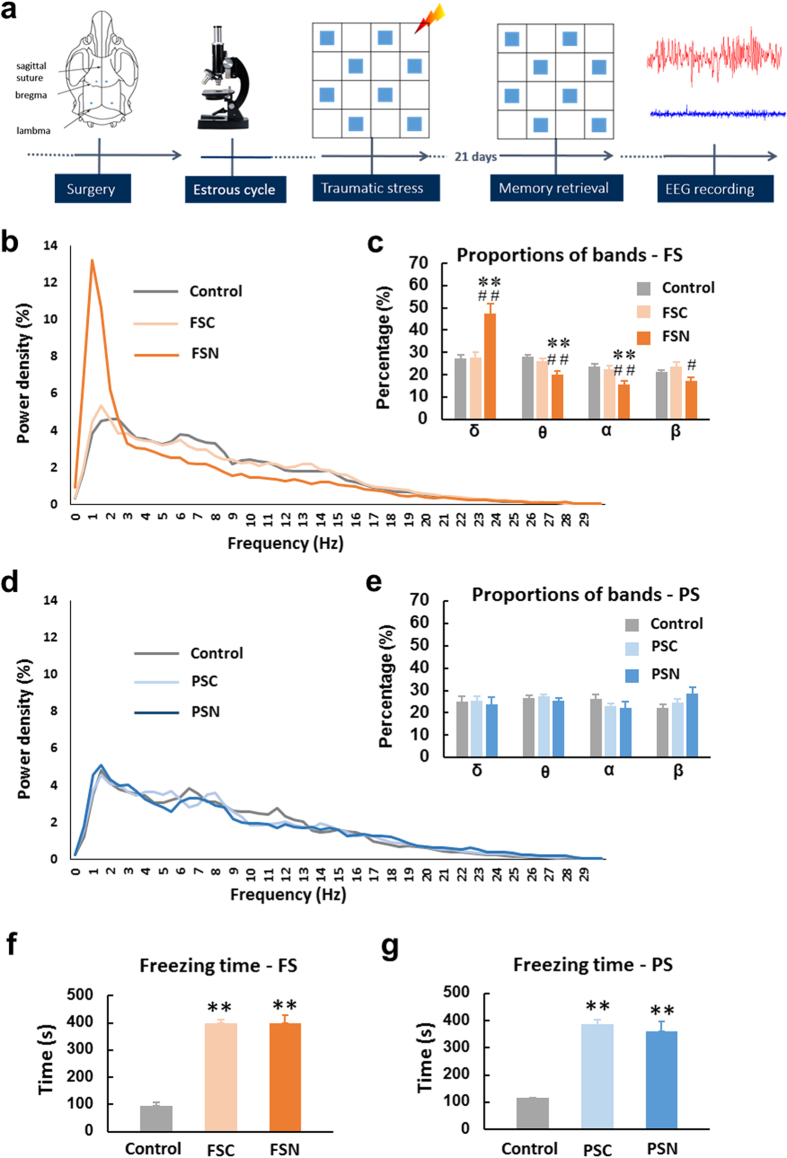
Rats presented startled awakening 21 days after traumatic stress. (**a**) Experimental procedure for cortical EEG analysis. Female Sprague-Dawley rats were implanted with electrodes for sleep recording. After 7 days of recovery, the rats were placed in the communication box and subjected to footshock or psychological stress in the proestrous phase. Twenty-one days after stress exposure, to evoke trauma-related memory, the rats were returned to the communication box in the absence of footshock, and sleep was recorded for 6 h. (**b,d**) EEG power density of 1-min sleep epoch immediately before startled awakening and normal awakening. *n* = 10–24 per group. Proportions of (**c,e**) delta (δ, 0–4 Hz), theta (θ, 4–8 Hz), alpha (α, 8–12 Hz), and beta (β, 12–30 Hz) bands. **p < 0.01, different from control group; ^#^*p* < 0.05 and ^##^*p* < 0.01, different from FSC group. *n* = 10–14 per group. (**f,g**) When the rats were returned to the communication box, the freezing duration significantly increased in the FS and PS groups compared with the control group. **p < 0.01, different from control group (Student-Newman-Keuls test). The data are expressed as mean ± SEM.

During sleep monitoring, we observed an extreme behavioral phenomenon that was distinguished from normal awakening (Video S1, *n* = 113), in which 16.74% (37/221) of FS rats (Video S2) and 15.58% (24/154) of PS rats (Video S3) exhibited startled awakening. We further analyzed the cortical EEG of 1 min before rats came to startled awakening or normal awakening. The EEG analysis indicated that FS-induced startled awakening (FS-induced “nightmare” [FSN])” was associated with the feature of higher delta power density (*F*_2,39_ = 15.822, *p* < 0.01) and lower theta power density (*F*_2,39_ = 11.057, *p* < 0.01), alpha power density (*F*_2,39_ = 10.721, *p* < 0.01) and beta power density (*F*_2,39_ = 4.124, *p* < 0.05; Fig. 1b,c). In contrast, the power spectra of the cortical EEG just before the PS-induced startled awakening (PS-induced “nightmare” [PSN]) were not altered (Fig. 1d,e). Both FSNs (*F*_2,161_ = 61.900, *p* < 0.01; Fig. 1f) and PSNs (*F*_2,122_ = 40.799, *p* < 0.01; Fig. 1g) exhibited a long freezing duration when they were returned to the communication box 21 days after traumatic stress.

### Remote memories were retrieved in different neocortical subregions in FSN and PSN rats

#### c-Fos distribution in somatosensory cortex

**Figure 2:**
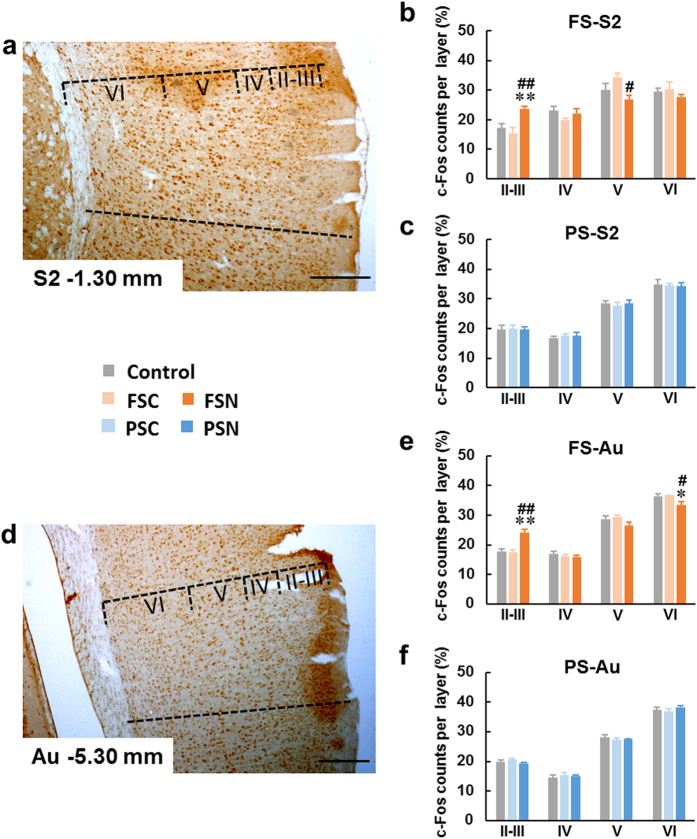
S2 and Au neocortex were involved in remote memory retrieval when FSNs presented startled awakening. (**a,d**) Photomicrographs of Fos staining in S2 and Au cortical layers II-VI after normal and startled awakening, respectively. Scale bars = 200 μm. (**b**) In the FSN group, Fos-positive neurons in S2 increased in layers II-III and decreased in lay V. (**c**) The PSN group did not exhibit a significant difference in S2. (**e**) In the FSN group, Fos-positive neurons. (**f**) Increased in layer II-III of Au, and decreased in lay VI. The PSN group did not exhibit a significant difference in Au. The data are expressed as mean ± SEM. *p < 0.05, **p < 0.01, different from control; ^#^*p* < 0.05, ^##^*p* < 0.01, different from FSC, *n* = 6–22 per group. S2, secondary somatosensory cortex; Au, primary auditory cortex.

Considering that FS- and PS-related remote traumatic memories may be associated with somatic sensations, especially in FS rats, we detected c-Fos immunoreactivity in the somatosensory cortex. The c-Fos distribution in the primary somatosensory cortex (S1FL, S1HL, and S1J) did not show significant changes in either the FSN or PSN group (Fig. S3). Compared with the control group and FS control (FSC) group, the distribution of c-Fos-positive neurons in secondary somatosensory cortex (S2) increased in superficial layers II-III (*F*_2,15_ = 8.210, *p* < 0.01) and decreased in deep cortical layer V (*F*_2,15_ = 5.177, *p* < 0.05) in FSN rats (Fig. 2b S2). No significant changes were observed in the PSN group (Fig. 2c).

#### c-Fos distribution in auditory cortex

In the communication box experiments, the electric footshock caused the FS rats to scream, and both the FS rats and PS rats could hear this sound. Therefore, we detected c-Fos distribution in the primary auditory cortex (Au), which is directly implicated in the storage of specific information about auditory experiences^15^. Compared with the control and FSC groups, the distribution of c-Fos-positive neurons increased in layers II-III of the Au (*F*_2,51_ = 12.60, *p* < 0.01) and decreased in layer VI (*F*_2,51_ = 4.456, *p* < 0.05) in FSN rats (Fig. 2e, S2). However, there was no significant changes in PSN rats (Fig. 2f, S2).

#### c-Fos distribution in visual cortex

Dreaming is a subjective experience during sleep that often has vivid visual content^16^. We detected c-Fos distribution in cortical regions that are responsible for visual imagery and perception, including the monocular area of the primary visual cortex (V1M), binocular area of the primary visual cortex (V1B), mediomedial area of the secondary visual cortex (V2MM), mediolateral area of the secondary visual cortex (V2ML), and lateral area of the secondary visual cortex (V2L). The c-Fos distribution in the primary visual cortex (V1B and V1M) showed no significant changes in either the FSN or PSN group (Fig. S4). No apparent signs of changes in activation were found in the secondary visual cortex (V2MM, V2ML, and V2L; Fig. S5).

#### c-Fos distribution in rhinal cortex

The rhinal cortex is composed of the ectorhinal (Ect), perirhinal, and entorhinal (Ent) subareas and recruited during stressful situations^17^. It has also been found to mediate long-term memory representations in several animal species, including humans^18^. In the present study, the distribution of c-Fos-positive neurons in the lateral Ent (LEnt, bregma −5.20 mm; Fig. S6) showed no significant changes in either the FSN or PSN group. Likewise, the Ect in three different coronal planes (bregma −4.52 mm, −5.20 mm, and −6.72 mm) also showed no significant changes in the FSN and PSN groups and their respective control groups (Fig. S7).

#### c-Fos distribution in association cortex

**Figure 3:**
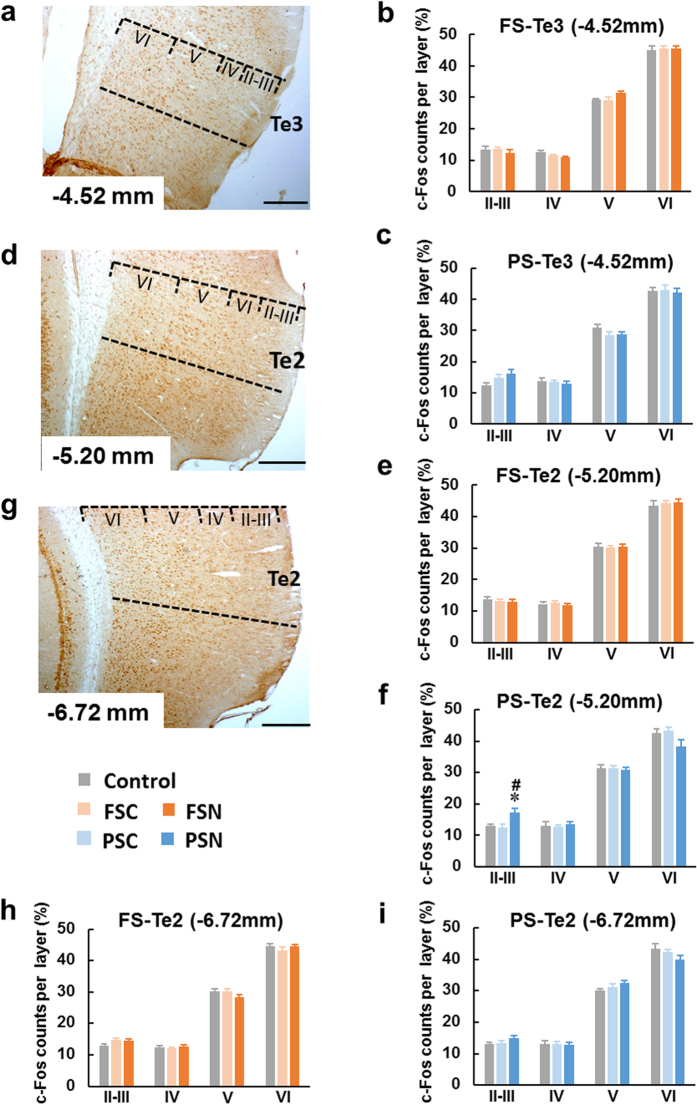
Te2 (bregma −5.20 mm) neocortex was involved in remote memory retrieval when PSNs presented startled awakening. (**a,d,g**) Photomicrographs of c-Fos staining in Te (bregma −4.52, −5.20, and −6.72 mm) cortical layers II-VI after normal and startled awakening, respectively. Scale bars = 200 μm. (**b,c**) The FSN and PSN groups did not exhibit significant differences in Te3 (bregma −4.52 mm). (**e**) The FSN group did not exhibit a significant difference in Te2 (bregma −5.20 mm). (**f**) In PSN rats, Fos counts significantly increased in layers II-III of Te2 (bregma −5.20 mm) compared with the control and PSC groups. (**h,i**) The FSN and PSN groups did not exhibit significant differences in Te2 (bregma −6.72 mm). The data are expressed as mean ± SEM. *p < 0.05, different from control; ^#^*p* < 0.05, different from PSC. *n* = 6–7 per group. Te, temporal association cortex.

The temporal association cortex 2 (Te2) is a multimodal area that subserves both auditory and visual sensations^19^. The analysis of c-Fos expression in Te2 (bregma −5.20 mm) revealed that layers II-III were significantly activated in the PSN group (*F*_2,15_ = 5.782, *p* < 0.05; Fig. 3f, S8). No changes in activity were observed in the other groups, including the FSN group (Fig. 3e). The other coronal planes of the temporal association cortex (bregma −4.52 mm and −6.72 mm) showed no changes in activation in the FSN and PSN groups (Fig. 3).

The posterior parietal/anteromedial cortex in rodents, also known as the parietal association cortex (PtA), plays an important role in perceptual memory, long-term memory encoding and consolidation, and the retrieval of spatial information^20^. We did not detect any changes in c-Fos expression in any layer of the PtA in any of the groups (Fig. S9).

### Fear emotion evocation circuits in FSN and PSN rats

#### c-Fos expression in amygdala

**Figure 4:**
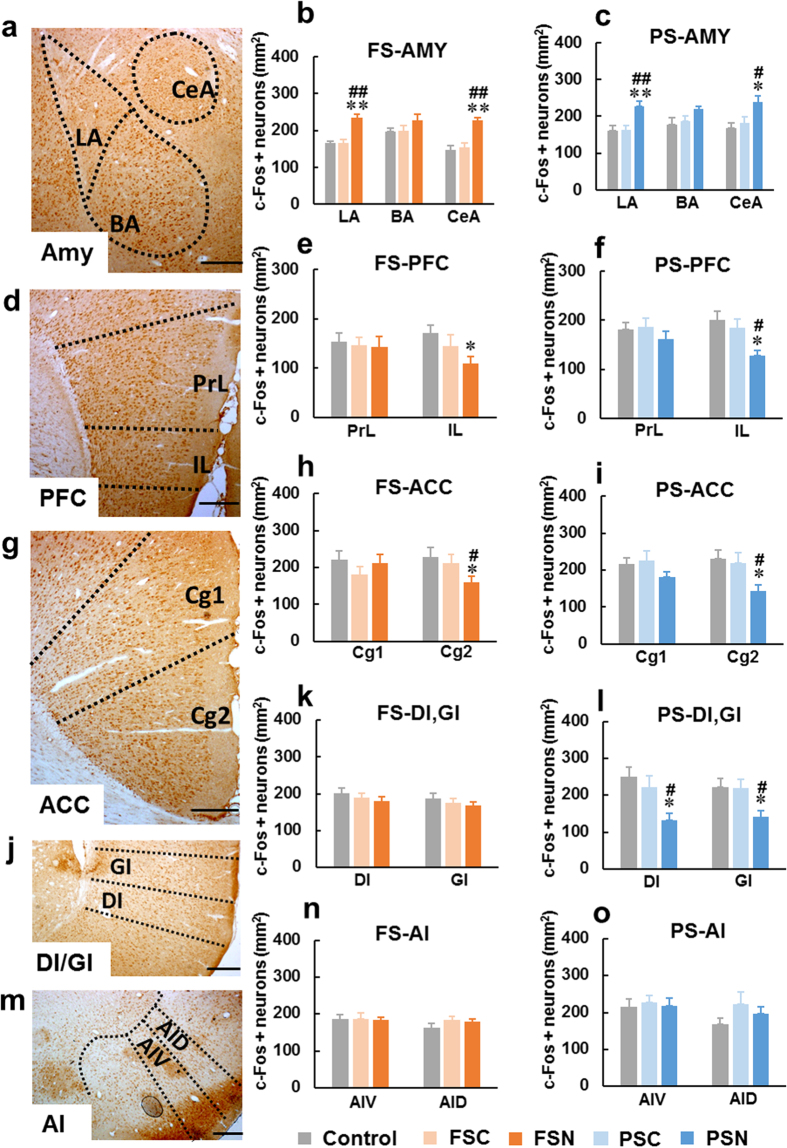
Fear emotion was evoked in FSN and PSN rats. (**a,d,g,j,m**) Photomicrographs of Fos staining in the amygdala, prefrontal cortex, anterior cingulate cortex, DI/GI subregions of the insula, and AI subregions of the insula, respectively. Scale bars = 200 μm. (**b,c**) Activity in the LA and CeA but not BA was significantly enhanced when FSN and PSN rats were startled awake. (**e,f**) Activity in the IL decreased in FSN and PSN rats, whereas the PrL showed no significant changes. (h, i) Cg2 activity decreased in FSN and PSN rats, whereas the Cg1 showed no significant changes. (**k,n**) c-Fos expression in regions of the insular cortex (AIV, AID, DI, and GI) did not change in FS and FSN rats. (**l,o**) The PSN group exhibited a significant decrease in c-Fos expression in the DI and GI compared with the PSC group; no significant changes were observed in the AIV and AID. The data are expressed as mean ± SEM. *p < 0.05, **p < 0.01, different from control; ^#^*p* < 0.05, ^##^*p* < 0.01, different from FSC/PSC (Student-Newman-Keuls test). *n* = 6–10 per group.

In complex vertebrates, including humans, the amygdala plays a primary role in the formation and storage of memories that are associated with emotional events. The amygdala consists of several anatomically and functionally distinct nuclei, including lateral (LA) and basal (BA) nuclei (together referred to as the basolateral amygdala [BLA]) and central nucleus of the amygdala (CeA)^21^,^22^. Compared with the control, FSC, and PS control (PSC) groups, c-Fos expression in the LA increased significantly in both the FSN (*F*_2,27_ = 28.452, *p* < 0.01) and PSN (*F*_2,21_ = 8.056, *p* < 0.01) groups. c-Fos expression in the CeA also increased significantly in both the FSN (*F*_2,27_ = 17.517, *p* < 0.01) and PSN (*F*_2,21_ = 5.154, *p* < 0.05) groups. c-Fos expression in the BA was not significantly different among groups (Fig. 4a–c, S10).

#### c-Fos expression in prefrontal cortex

The mPFC is a cortical structure that is composed of several nuclei, including the infralimbic (IL) and prelimbic (PrL) cortices. A growing body of evidence indicates that the IL and PrL work in a specific and opposite manner^23^,^24^,^25^. c-Fos expression decreased in the IL in both the FSN (*F*_2,27_ = 3.905, *p* < 0.05) and PSN (*F*_2,18_ = 5.027, *p* < 0.05) groups, but no significant differences were found in the PrL (Fig. 4d–f, S11).

#### c-Fos expression in anterior cingulate cortex

The ACC is divided into two parts: a dorsal part (Cg1) that is involved in attention and cognitive control and a ventral part (Cg2) that is involved in emotional regulation^25^,^26^. In the present study, c-Fos expression in the Cg2 decreased in both the FSN (*F*_2,25_ = 3.897, *p* < 0.05) and PSN (*F*_2,24_ = 4.161, *p* < 0.05) groups, whereas Cg1 activity did not change (Fig. 4g–i, S12).

#### c-Fos expression in insular cortex

The insular cortex has granular (GI), dysgranular (DI), and agranular (AI) subregions in rats. The insular cortex has connections with limbic structures, including the amygdala, thalamus, and mPFC^27^,^28^, suggesting that it may be part of fear and anxiety circuitry in the brain^29^. We detected c-Fos expression in the GI, DI, AI dorsal part (AID), and AI ventral part (AIV) to investigate whether the insula is involved in startled awakening. The PSN group exhibited significant decreases in Fos expression in the GI (*F*_2,18_ = 5.594, *p* < 0.05) and DI (*F*_2,24_ = 4.339, *p* < 0.05; Fig. 4j–l, S14). No significant changes were observed in the AID or AIV (Fig. 4m–o). We also did not observe changes in Fos expression in the GI, DI, AID, or AIV in the PSC, FSC, or FSN group (Fig. 4j–o, S13).

#### c-Fos expression in habenula and paraventricular thalamic nucleus

The habenula is traditionally divided into lateral (limbic) and medial (motor) parts. Neurons in the lateral habenula (LHb) are “reward-negative” because they are activated by stimuli that are associated with unpleasant events^30^. We did not observe any changes in the activation of the lateral part or medial part of the LHb in any of the groups (Fig. S14).

The PVT receives multiple inputs from the brainstem and hypothalamus and targets the mPFC and amygdala^31^,^32^. A recent study indicated that the PVT may act as a crucial thalamic node that is recruited to PrL-CeA networks for the retrieval and maintenance of long-term auditory conditioned fear memories^33^. We did not observe significant changes in c-Fos expression in the PVT in either the FSN (*F*_2,18_ = 1.414, *p* = 0.270) or PSN (*F*_2,18_ = 0.300, *p* = 0.744) group (Fig. S14).

#### c-Fos expression in motor cortex

**Figure 5:**
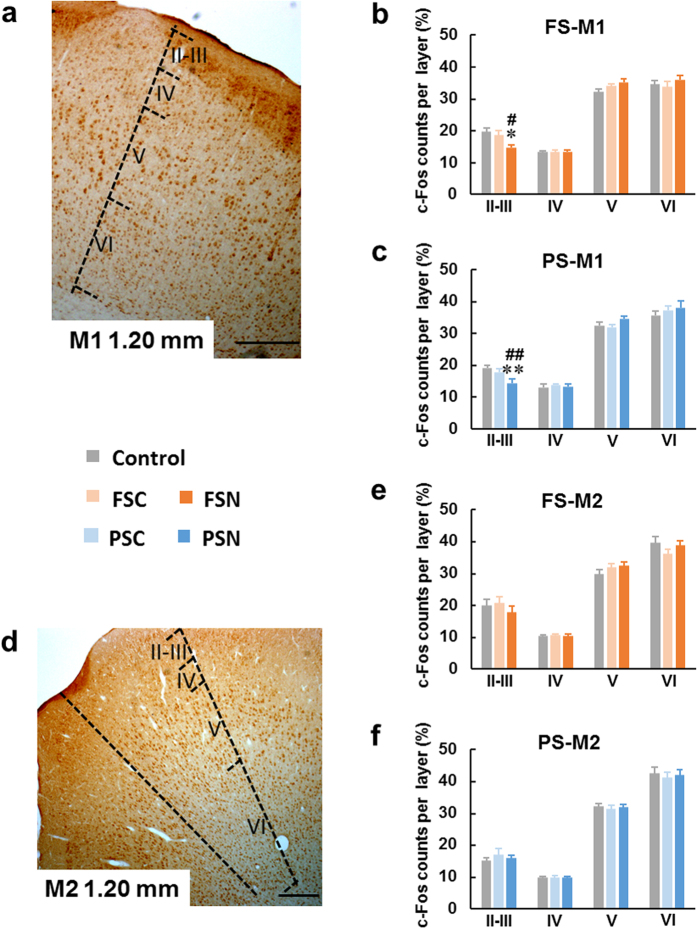
c-Fos distribution in primary and secondary motor cortex. (**a,d**) Photomicrographs of c-Fos staining in M1 and M2, which control forelimb movements. Scale bars = 200 μm. (**b,c**) After FSN and PSN rats were startled awake, c-Fos counts significantly decreased in layers II–III of M1 compared with rats that awoke normally. (**e,f**) c-Fos protein expression was not different among groups in M2. The data are expressed as mean ± SEM. *p < 0.05, **p < 0.01, different from control; ^#^*p* < 0.05, ^##^*p* < 0.01, different from FSC/PSC (Student-Newman-Keuls test). *n* = 6–8 per group. M1, primary motor cortex; M2, secondary motor cortex.

Startled awakening is a type of abnormal awakening. In the present study, it was associated with jumping behavior during sleep (Video S1-S3). Such behavior is undoubtedly associated with the motor cortex. Therefore, we tracked c-Fos expression in the primary motor cortex (M1) and secondary motor cortex (M2)^34^. We observed a marked decrease in c-Fos expression in layers II-III of M1, which is responsible for forelimb movements^35^, in both the FSN (*F*_2,18_ = 4.637, *p* < 0.05) and PSN (*F*_2,21_ = 19.34, *p* < 0.01) groups (Fig. 5a–c, S15). No significant changes were observed in the M2 subregion in either the FSN or PSN group (Fig. 5d–f).

## Discussion

**Figure 6:**
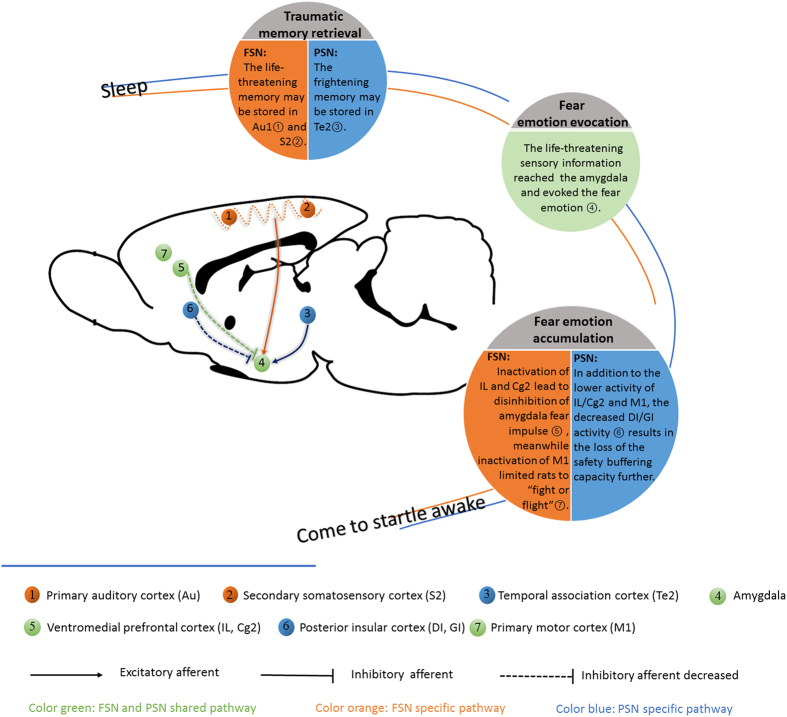
The presumed neuroanatomy of FS and PS rats that are startled awake. When rats experience traumatic “nightmares,” trauma-related memory that is stored in the neocortex is retrieved. In FSN rats, the traumatic memory is stored in S2 and Au. In PSN rats, the traumatic memory is stored in Te2. The traumatic memory arouses fear emotion, reflected by enhanced activity in the amygdala. The suppression of activity in the vmPFC (including the IL and Cg2) fails to inhibit the hyperfunction of the amygdala (LA and CeA), resulting in the accumulation of fear emotion. Suppression of the posterior insular cortex (DI and GI) may also be a factor that contributes to the accumulation of fear emotion, peculiarly in PSN rats. A decrease in M1 activity might also result in inhibition of the rats’ ability to express “fight or flight” reactions, thus placing the rats in an “emergency situation” in a traumatic nightmare. As a result, rats present startled awakening from a “nightmare”.

Posttraumatic nightmares, as a core component of posttraumatic stress disorder^36^,^37^, are frequently associated with the female gender, increased stress, psychopathology, and dispositional traits^4^. Such nightmares are clinically relevant in today’s relatively violent world^2^,^3^,^4^. Its pathogenesis has remained unexplained, including differences between traumatic events that are experienced and witnessed and psychological trauma. Objective parameters that are associated with nightmares need to be identified to better understand the underlying mechanisms of nightmares in PTSD and develop possible therapeutic strategies. The current animal models, like footshock stress, underwater, predators, can mimic the core aspects of human PTSD, including individual differences in susceptibility to various stressors. In the present study, female rats received two types of stress (FS and PS), and both types of stress elicited startled awakening. The majority of startled awakening events happened during 3–6 hour from the start of sleep recording (Fig. S16), in this case, the direct influence of re-exposed to communication box on c-Fos expression would be very faint^38^, which was reflected in the c-Fos expression in the FSC and PSC groups. Accordingly, these results implied that such remote memory retrieval and negative emotion evocation that appeared in FSNs and PSNs could be recognized as the fear memory retrieval during sleep. Since the startled awakening was related to such fear memory retrieval and occurred only in rats exposed stress, we speculate that startled awakening might mimic posttraumatic nightmares. The current results identified common neural pathways, also suggested mechanisms that may distinguish between FS and PS-induced startled awakening (Fig. 6). Both FSNs and PSNs were accompanied by remote memory retrieval and fear emotion evocation when rats came to startled awakening, but the neurocircuitry of traumatic memory storage and retrieval and the pathway that consolidates fear emotion memory were different between the FSN and PSN groups.

The representation of the memory of a traumatic experience in a dream is a distinguishing feature of PTSD. Such nightmares replicate the actual memory of the traumatic experience and are associated with the development of PTSD^39^. Remote emotional memory storage and retrieval are associated with the cortico-cortical connections. Layers II and III of neocortex are the origin and termination of most cortico-cortical connections. Immunoreactivity of c-Fos was pronounced in more superficial layers II-III following the remote memory test^10^,^11^,^12^,^13^. Here we assessed c-Fos distribution in II-VI layers of cortical regions, and the increased distribution of c-Fos-positive neurons in superficial layers was applied as a method to assess whether the interested cortical regions were recruited by the online remote memory. The present results indicated that when rats exhibited startled awakening, the remote memories stored in different cortical subregions may be recalled (Figs 2 and 3). In the FSN rats, the memory may be retrieved from S2 and Au, which may be because FSN rats received the electric FS directly and screamed. In PSN rats, when they presented startled awakening, the remote memory which stored in Te2 was online, Te2 is an “association” cortex, a part of the visual association cortex, and contributes to the caudal part of the auditory belt cortex^19^. Te2 has been reported to play a role in the acquisition and retrieval of recognition memory^40^,^41^ and it was also considered as a remote auditory fear memory storage region^12^, even so, this was an unexpected result. We presume that at the moment when PSN rats perceived themselves to be in a dangerous situation, Te2 of the association cortex processed and integrated the complex psychological information. Many of the structures and functions of subcortical nuclei are conserved across species, from rodents to primates. However, the neocortex, particularly the association cortex which is considered crucial for higher cognitive and behavioral functions, extensively expanded in primates as a result of evolutionary pressure^42^,^43^. Situations in which humans experience traumatic stress are more complex, and we speculate that the association cortex, such as Te2, plays a crucial role in some PTSD patients.

The present results indicated that when FSN and PSN rats presented startled awakening from different cortical electrical activities. Unlike PSN rats, FSN rats were startled awake from high delta power (slow wave) sleep, in FSN group, the proportions of delta band was 47.4% (Fig. 1c), however, in PSN rats, the proportion of delta band was 23.7% (Fig. 1e). Slow-wave activity during sleep is associated with synaptic downscaling and the nullification of weak potentiation, thus improving the signal/noise ratio. Consequently, synapses with strong connections become relatively prominent^44^. We speculated that in FSN rats before they presented startled awakening, the cortices may be immersed in slow-wave activity, and synapses with relatively strong connections may exist in S2 and Au. In turn, these subareas may participate in the remote physiological traumatic memory storage circuit. In PSN rats, remote psychological traumatic memories may have been retrieved differently and caused startled awakening from a stage of sleep with a power density of the EEG spectrum that was similar to rats that awoke normally.

Compared with other dreams, posttraumatic nightmares are associated with more generally negative emotions, such as panic, fear, and threat, and these appear to be most pronounced in PTSD^14^. As expected, compared with normal awakening, activity in both the LA and CeA significantly increased in the FSN and PSN groups, whereas activity in the BA did not significantly change. The increase in activity in the LA and CeA suggests that when the rats were startled awake, fear emotions were evoked. The amygdala plays a pivotal role in triggering a state of fear (especially the BLA) and expressing the behavioral fear response (especially the CeA)^21^. A previous study^12^ and the present results support a role for the amygdala in the retrieval of remote fear memory. The present results suggest that portions of the mPFC (including the IL and Cg2) were hyporesponsive (Fig. 4e,f) and may account for the deficit of fear extinction and exaggerated fear responses^21^,^22^. A previous study found that inactivation of the IL but not PrL impaired the consolidation and retrieval of fear extinction^24^. The IL plays a role in fear extinction by inhibiting activity in the amygdala either directly or indirectly^24^,^25^. Negative functional connectivity between the supragenual ACC (Cg2 in the rat) and amygdala has been reported^26^. Lower activation in the Cg2 and IL in FSN and PSN rats in the present study was consistent with a previous report that found that individuals with interpersonal violence-related PTSD, compared with healthy controls, had lower activity in the ventral ACC (Cg2) and mPFC during all emotional scenes compared with neutral scenes^45^. Morphometric studies indicated that reductions of the volume of the ventral ACC and vmPFC were common in anxiety disorder^46^. At the same time, rats fell into immobility because the decreased M1 activity (Fig. 5), FSN and PSN rats share the above pathways. In the present study, PSN rats appeared to recruit an additional pathway, in which activity in the DI and GI also decreased, thus contributing to the accumulation of fear emotion. The DI and GI are referred to as the posterior insular cortex or “sensory insula”^29^, play critical role in safety learning and mediate the stress-buffering effects of safety signals^47^. In concert with the amygdala, the sensory insula may play a critical role in fear inhibition^48^. Based on our findings, we may speculate that restrained activity in the DI and GI led PSN rats to lose their safety buffering potential, with accelerated fear emotion accumulation in the amygdala that consequently resulted in “nightmares” in PSN rats. However, our results cannot explain why FSN and PSN rats exhibited different changes in activity in the sensory insula.

Replication of the actual traumatic memory and negative emotion evocation are common features in PTSD-like nightmares. Emotional events often attain a privileged status in memory. Fear is a powerful emotion, and the memories associated with fear-provoking events are robust. Although dreams can potentially have a positive influence on emotional adaptation^49^, recurring nightmares in PTSD patients reinforce the traumatic memory and contribute to the dreamer’s fear of traumatic events. Identifying the relationships between affective and memory processes and clarifying the underpinnings of these relationships can provide novel directions for exploring the mechanisms of PTSD-like nightmares.

An important factor of trauma in humans is the perception of the life-threatening potential of the situation^1^. It is unclear whether such stress can be regarded as a life-threatening traumatic event in rats and which stressors will be most effective in rats. Also, we have no idea that whether or not the startled awakening would happened in rats exposed to other kinds of stress except FS or PS. Another limitation of the present study was that we cannot unequivocally state that the rats were actually experiencing a traumatic memory before being startled awake. Nevertheless, startled awakening may still be regarded as a possible animal model that mimics symptoms of recurring nightmares in individuals with PTSD. This extreme phenomenon may offer a unique opportunity to elucidate the emotional and memory processing of posttraumatic nightmares. Understanding the mechanisms of posttraumatic nightmares may open new possibilities for developing innovative treatments for PTSD nightmares.

## Materials and Methods

### Animals

Female Sprague-Dawley rats (250–270 g, Grade I, purchased from the Animal Center of Peking University, Beijing) were housed individually in plastic cages and maintained under an artificial 12 h/12 h light/dark cycle (lights on 8:00 AM to 8:00 PM) at 23 ± 1 °C and 50 ± 10% humidity. The rats had *ad libitum* access to food and water. All of the experiments were conducted in accordance with the European Communities Council Directive (2010/63/EU) for the use of experimental animals and approved by the Peking University Committee on Animal Care and Use.

### Communication box

The communication box (64 cm × 64 cm × 8 cm) was equipped with a grid floor that was composed of stainless steel rods (0.5 cm diameter) placed 1 cm apart. The box consisted of 16 compartments (16 cm × 16 cm × 8 cm) that were separated by transparent plastic plates. Rats in eight compartments were exposed to an electrified grid through which electric FSs were delivered. The other eight compartments had plastic plates (8 cm × 8 cm) on top of the grid floor to prevent the rats from receiving the electric shocks. The rats that were placed in the compartments with the electric grid floors comprised the FS group. The rats that were placed in the compartments with the plastic plates comprised the PS group. The PS group was exposed to visual, olfactory, and auditory stimuli (e.g., struggling, vocalizing, defecating, urinating, and jumping) that were emitted by FS rats (Fig. S1a).

### Experimental procedure

Electrodes were implanted to monitor sleep. At least 7 days after electrode implantation, the rats were introduced to the communication box to receive FS or PS in the proestrous phase. Twenty-one days after stress exposure, to evoke trauma-related memory, the rats were returned to the communication box in the absence of electric shocks. Freezing behavior was recorded, which can reflect the degree of emotional fear and intensity of traumatic memory when rats are reexposed to a trauma cue. After that, the rats were moved to the sleep box which is an electrically shielded room and noise-attenuated environment free from interruptions. We then recorded the rats’ EEG sleep patterns for 6 h and monitored whether they presented startled awakening, defined as the FSN and PSN groups (Fig. 1a). The rats were sacrificed immediately after they were startled awake and perfused for neural circuitry exploration. The FSC and PSC groups consisted of rats that did not present startled awakening. The control group (see below) and FSC and PSC groups were sacrificed immediately after normal awakening.

### Estrous cycle determination

Vaginal swabs and cycle phase assessment were conducted between 9:00 AM and 10:00 AM at least 5 consecutive days prior to exposure to FS and PS. Vaginal secretions were collected by inserting the tip of a plastic pipette, filled with 10 μl of physiological saline, into the rat’s vagina. One drop of the smear was collected with a clean tip from each rat. The vaginal fluid was then placed on glass slides. The samples were examined under a light microscope fitted with 8 ×  and 15 × objective lenses. The estrous cycle consists of four different phases: diestrus, proestrus, estrus, and metestrus^50^.

### Footshock and PS established an animal model of PTSD

The rats were randomly divided into three groups: control group (exposure to communication box without any stress, the rats of control for FS group were put in the foot-shock compartment and the control for PS group were put in the compartments which has plastic plate), FS group (exposure to FS stress), and PS group (exposure to psychological stress). The experiment was conducted once between 9:00 AM and 11:00 AM in a separate quiet room. Rats in the proestrous phase were placed in the communication box. After 5 min adaption, rats were subjected to the stress for 50 min. The FS intensity was 2 mA over 10 trials, 2.5 mA over 10 trials, 3 mA over 10 trials, 3.5 mA over 10 trials, and 4 mA over 10 trials. The shock intensity increased every 10 min^51^. (Fig. S1b).

### Surgery

The rats were anesthetized with an injection of chloral hydrate (300 mg/kg, i.p.), and electrodes for EEG and electromyographic (EMG) recording were implanted. Two stainless steel screws attached to insulated wire were implanted in the skull over the frontal-parietal cortex to record the EEG. One was placed approximately 2 mm anterior and 2 mm to the right of bregma, and the other was placed approximately 3 mm posterior and 2 mm to the left of bregma. A ground electrode was placed between the other two electrodes, 3 mm lateral to midline. For EMG recording, a pair of wire electrodes was threaded through the nuchal muscles. These electrodes were attached to a miniature connector that was affixed to the skull with dental acrylic^52^.

After surgery, the rats were injected with antibiotics for 3 days and allowed to recover for 7 days prior to the experiments. For habituation, they were connected to the recording apparatus at least 1 day before sleep recording.

### Traumatic memory retrieval

To evoke trauma-related memory, the rats were returned to the communication box for 10 min in the absence of FS, 21 days after stress exposure. Freezing behavior was used as an index of memory. Freezing was defined as the lack of movement, with the exception of respiration. Videotapes were viewed by a blinded observer to evaluate freezing behavior.

### Observations of startled awakening and EEG recording

After trauma-related memory retrieval, the rats were observed with an infrared camera to determine whether they presented startled awakening; the EEG and EMG were monitored simultaneously. The recording sessions occurred between 10:00 AM and 4:00 PM.

For electrophysiological recording, a lightweight shielded cable was plugged into the connector on the rat’s head and attached to a counterbalanced swivel that permitted free movement. The signals were routed to an electroencephalograph (Model MP 150, BIOPAC Systems, Goleta, CA, USA). The signals were amplified and filtered (EEG, 0.5–30 Hz; EMG, 16–128 Hz), digitized at a sampling rate of 128 Hz, and recorded using AcqKnowledge software (BIOPAC Systems). The EEG/EMG recordings were analyzed using SleepSign 2.0 software^52^.

### Histological and immunohistochemical procedures

The rats were sacrificed immediately after startled awakening or normal awakening, designated as the “nightmare” (FSN and PSN) and respective control (FSC and PSC) groups, respectively. Under deep chloral hydrate anesthesia (300 mg/kg, i.p.), the rats were perfused with 500 ml of 4% paraformaldehyde in 0.1 M phosphate-buffered saline (PBS; pH 7.4) and then with 500 ml of 0.01 M PBS. Whole brains were immediately removed and postfixed in the same fixative at 4 °C for 24 h and then immersed in 30% sucrose in PBS (0.01 M, pH 7.4) at 4 °C for cryoprotection. The brains were then rapidly frozen on dry ice and cut into 20 μm coronal sections with a cryostat (Leica CM1850, Leica Microsystems UK, Milton Keynes, UK). Coronal sets of brain regions possibly related to trauma-related memory storage and fear emotion are listed in Table S2^53^. The sections were stored at -20 °C until staining.

Fos protein immunostaining was performed in sequential sections using diaminobenzidine (DAB) as the chromogen. The sections were washed in PBS, incubated in 0.3% H_2_O_2_ for 10 min, and washed with PBS (3 × 5 min). Antigen retrieval was conducted in citrate buffer (pH 6.0) using a microwave. After the sections returned to room temperature, they were immersed in PBS that contained 0.3% Triton X-100 for 10 min, followed by incubation with bovine serum albumin for 60 min. The sections were incubated in the appropriate primary antibody diluted in PBS that contained 1% bovine nonspecific serum and 0.1% Triton X-100 for 12–16 h at 4 °C. We chose an appropriate antibody that recognized Fos (1:100; sc-52, Santa Cruz Biotechnology, Santa Cruz, CA, USA). After washing in PBS (3 × 5 min), the sections were incubated with biotinylated goat anti-rabbit immunoglobulin G (Bio/IgG) for 40 min at room temperature, washed in PBS (3 × 5 min), incubated with streptavidin-horseradish peroxidase for 20 min, and reacted with DAB for 5 min. Fos-immunoreactive nuclei showed brown staining.

### Fos protein expression analysis

Photomicrographs of various brain areas from anatomically matched sections were captured using a charge-coupled device camera (Leica DC 300) and light microscope with a 10 × objective (Leica DMR; Leica Microsystems, Wetzlar, Germany). In the PFC (IL, PrL), amygdala (BA, LA, CeA), insula (AIV, AID, DI, GI), and lateral habenula (LHbM, LHbL), Fos-positive cells were calculated using ImagePro Plus 6.0 software with particle analysis for constant regions of interest for each brain area. Positive nuclei were detected automatically using a threshold based on the staining density and target size. As possible cortices related to traumatic memory storage, Fos-labeled neurons were counted in layers II-VI. Immunoreactive nuclei were counted bilaterally using at least three serial sections for each area. The data were then averaged to produce group means.

### Statistical analysis

The data are expressed as mean ± SEM. The data were analyzed using one-way analysis of variance (ANOVA) followed by the Student-Newman-Keuls *post hoc* test for multiple comparisons. Values of *p* < 0.05 were considered statistically significant. All of the Fos mapping results are presented in Table S3 and S4.

## Additional Information

**How to cite this article**: Yu, B. *et al*. Different neural circuitry is involved in physiological and psychological stress-induced PTSD-like “nightmares” in rats. *Sci. Rep*. **5**, 15976; doi: 10.1038/srep15976 (2015).

## Supplementary Material

Supplementary Information

Supplementary Video S1

Supplementary Video S2

Supplementary Video S3
