# Supplementary material for: Different neural circuitry is involved in physiological and psychological stress-induced PTSD-like “nightmares” in rats
Source: Sci Rep. 2015 Nov 4;5:15976. doi: 10.1038/srep15976 (PMC4632128; doi:10.1038/srep15976)
Supplement: Supplementary Information [file srep15976-s1.pdf]

Supplementary Materials for

Different neural circuitry is involved in physiological and psychological stress-induced PTSD-like "nightmares" in rats

Bin Yu, Su-Ying Cui, Xue-Qiong Zhang, Xiang-Yu Cui, Sheng-Jie Li, Zhao-Fu Sheng, Qing Cao, Yuan-Li Huang, Ya-Ping Xu, Zhi-Ge Lin, Guang Yang, Jin-Zhi Song, Hui Ding &Yong-He Zhang

Department of pharmacology, Peking University, School of Basic Medical Science, 38 Xueyuan Road, Beijing, 100191, China.

Correspondence and requests for materials should be addressed to Y.H. Z. (zhyh@hsc.pku.edu.cn)

This PDF file includes:

Figures S1 to S16

Tables S1 to S7

Captions for Videos S1 to S3

Other Supplementary Materials for this manuscript includes the following:

Videos S1 to S3

Figure S1 to S16

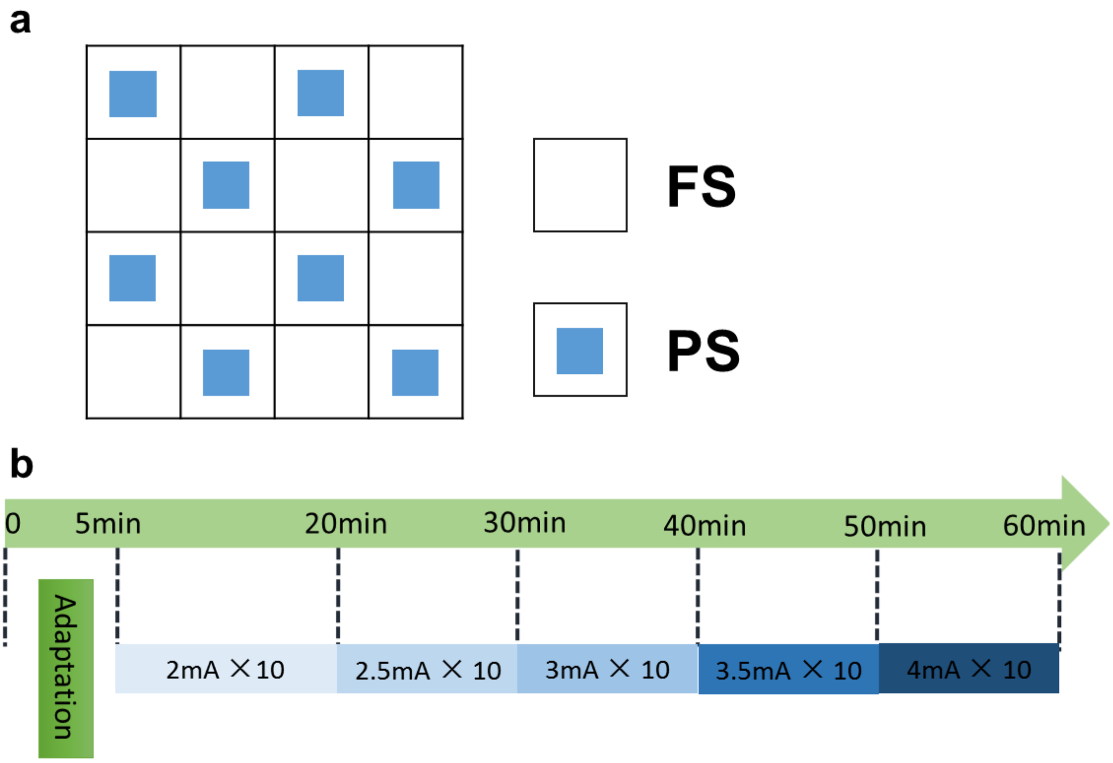

Figure S1. The schematic diagram of communication box and procedure of foot-shock. (**a**) The schematic diagram of communication box. Rats in the blank lattices exposed to the electrified grid directly, they received foot-shock stress (FS). While rats in the lattices with blue board could avoid FS, they exposed to psychological stress (PS) through receiving the visual, olfactory and auditory stimuli from physiological stress. (**b**) Procedure of foot shock and detailed electric parameters.

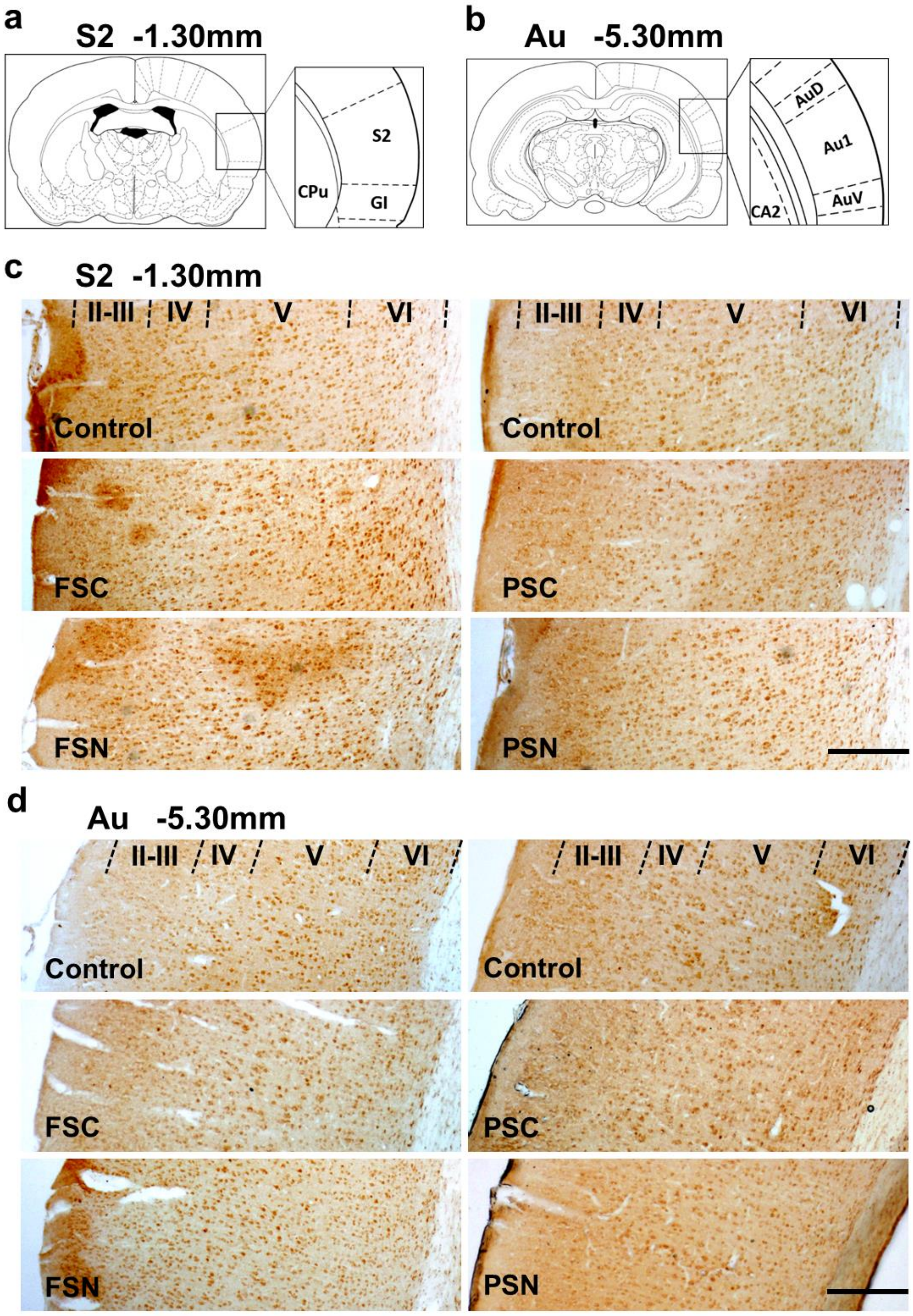

Figure S2. c-Fos distribution in FSN traumatic memory storage neocortex regions. (**a**, **b**) Illustrations of S2 (**a**) and Au (**b**) brain sections are based on the atlas of Paxinos and Watson. (**c**, **d**) Photomicrographs of c-Fos staining in S2 (**c**) and Au (**d**) cortical layers I-VI after startled awakening. The c-Fos distribution of FSN rats in the above regions was shift from deep layers to superficial layers. Scale bars = 200 μm.

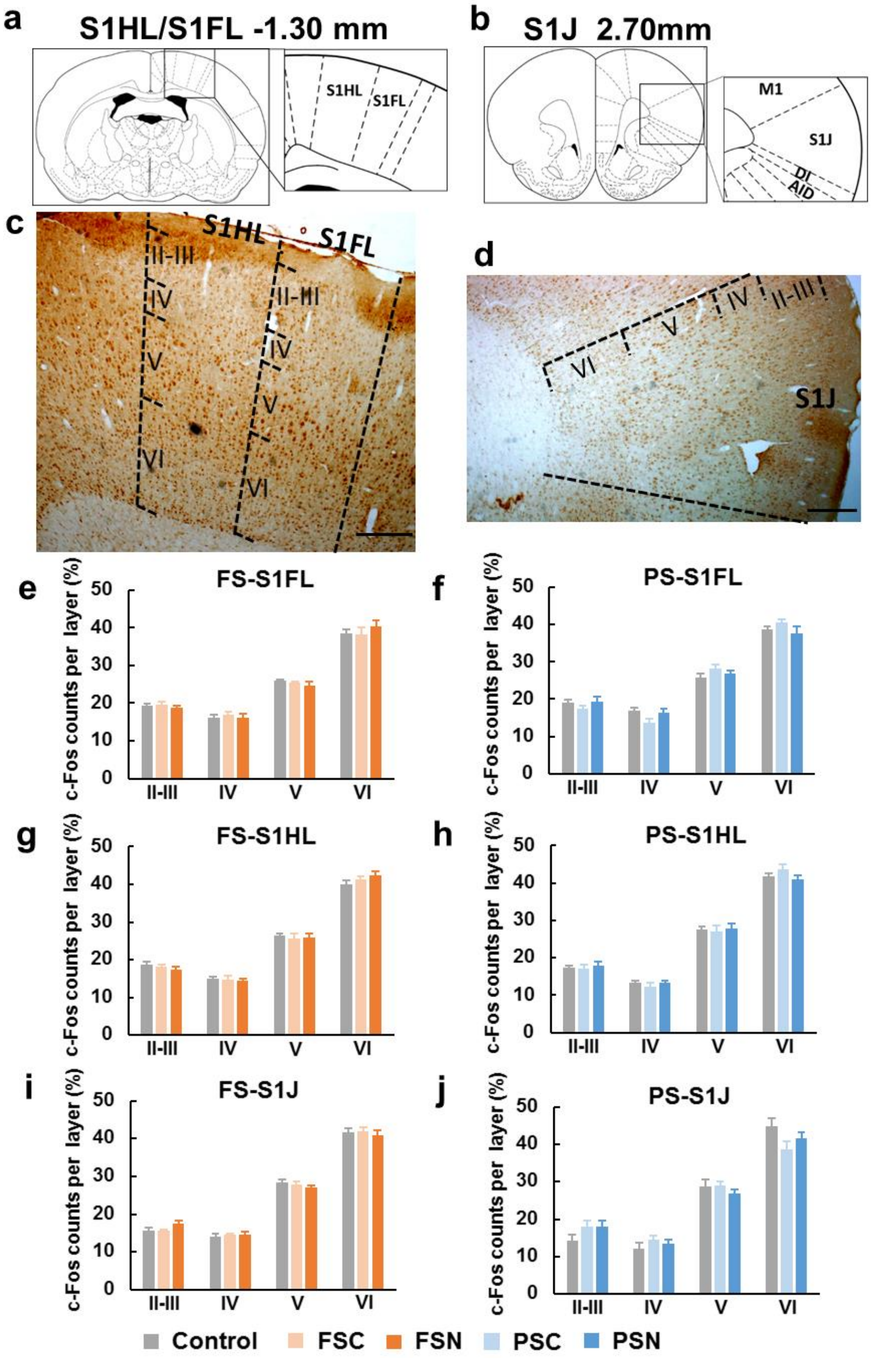

Figure S3. c-Fos expression in primary somatosensory cortex. (**a**) Illustrations of brain sections of S1 responsible for forelimb (S1FL) and hindlimb (S1HL). (**b**) Illustrations of brain sections of S1 responsible for jaw (S1J). (**c**) Photomicrographs of c-Fos staining in S1FL and S1HL. (**d**) Photomicrographs of c-Fos staining in S1J. (**e**, **f**) c-Fos protein analysis revealed no difference among groups in S1HL. Scale bars = 200 μm. (**g**, **h**) c-Fos protein analysis revealed no difference among groups in S1FL. (**i**, **j**) Fos protein analysis revealed no difference among groups in S1J. Data are represented as mean ± SEM. *n* = 6-7 per group. S1HL, Primary Somatosensory Cortex, hindlimb. S1FL, Primary Somatosensory Cortex, forelimb. S1J, Primary Somatosensory Cortex, jaw region.

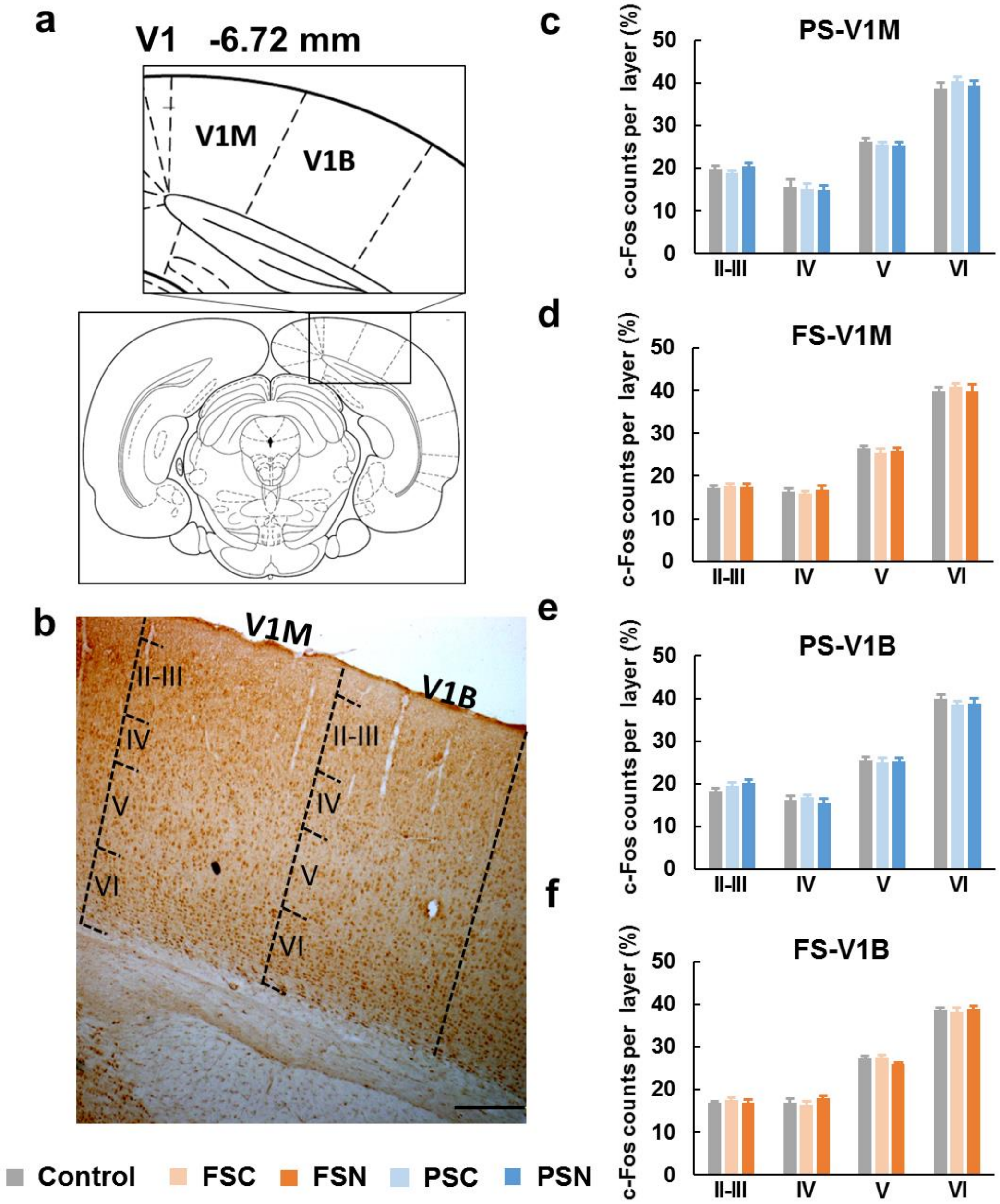

Figure S4. c-Fos expression in primary visual cortex. (**a**) Illustrations of brain sections of V1 binocular area (V1B) and monocular area (V1M). (**b**) Photomicrographs of c-Fos staining in V1B and V1M cortical layers I-VI. Scale bars = 200 μm. (**c**, **d**) c-Fos protein analysis revealed no difference among groups in V1M. (**e**, **f**) c-Fos protein analysis revealed no difference among groups in V1B. Data are represented as mean ± SEM. n = 7-8 per group. V1, primary visual cortex.

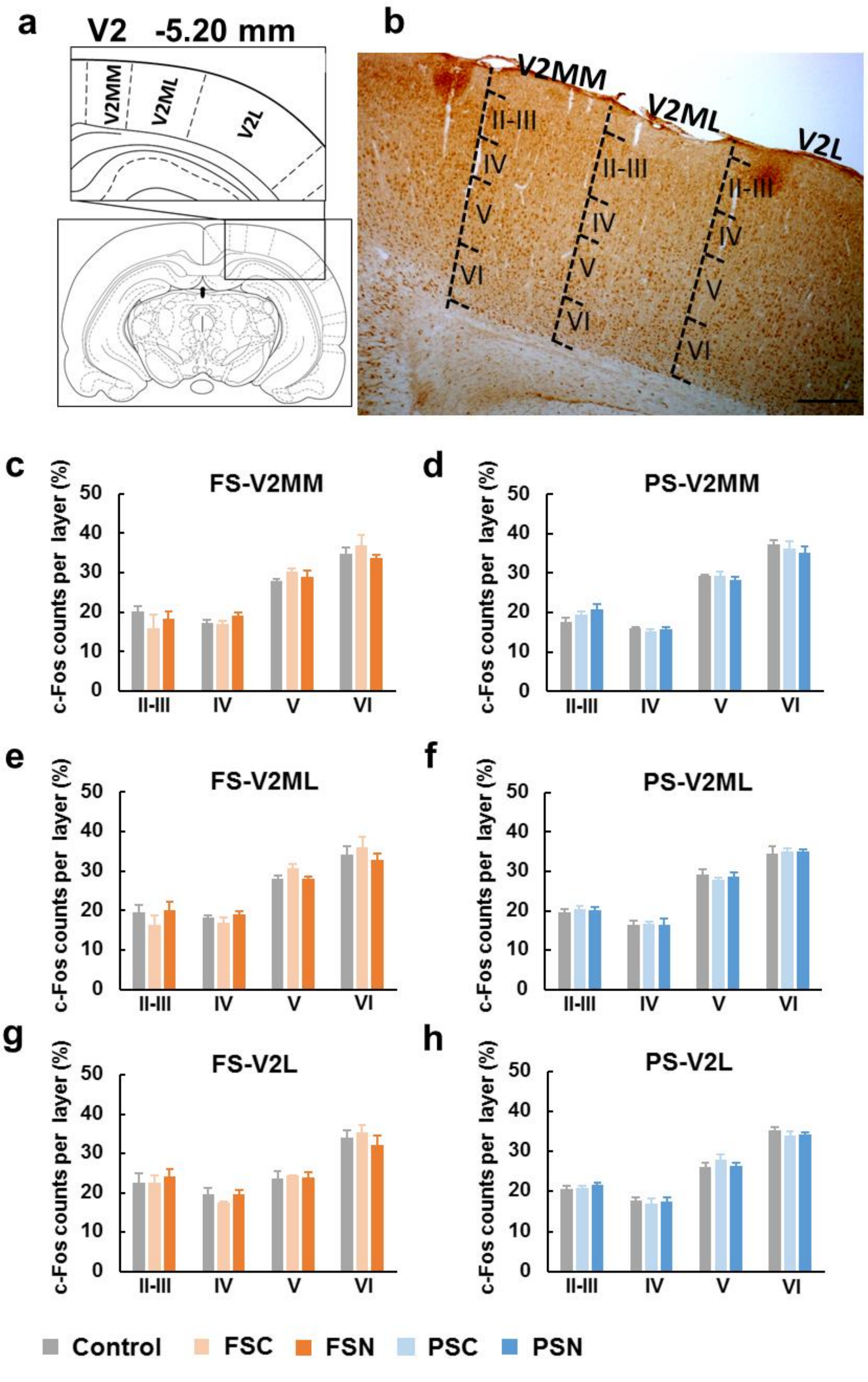

Figure S5. c-Fos expression in secondary visual cortex. (**a**) Illustrations of brain sections of V2 mediodial area (V2MM), mediolateral area (V2ML) and lateral area (V2L). (**b**) Photomicrographs of c-Fos staining in V2MM, V2ML and V2L cortical layers I-VI. Scale bars = 200 μm. (**c**, **d**) Fos protein analysis revealed no difference among groups in V2MM. (**e**, **f**) c-Fos protein analysis revealed no difference among groups in V2ML. (**g**, **h**) c-Fos protein analysis revealed no difference among groups in V2L. Data are represented as mean ± SEM. *n* = 5-6 per group. V2, secondary visual cortex.

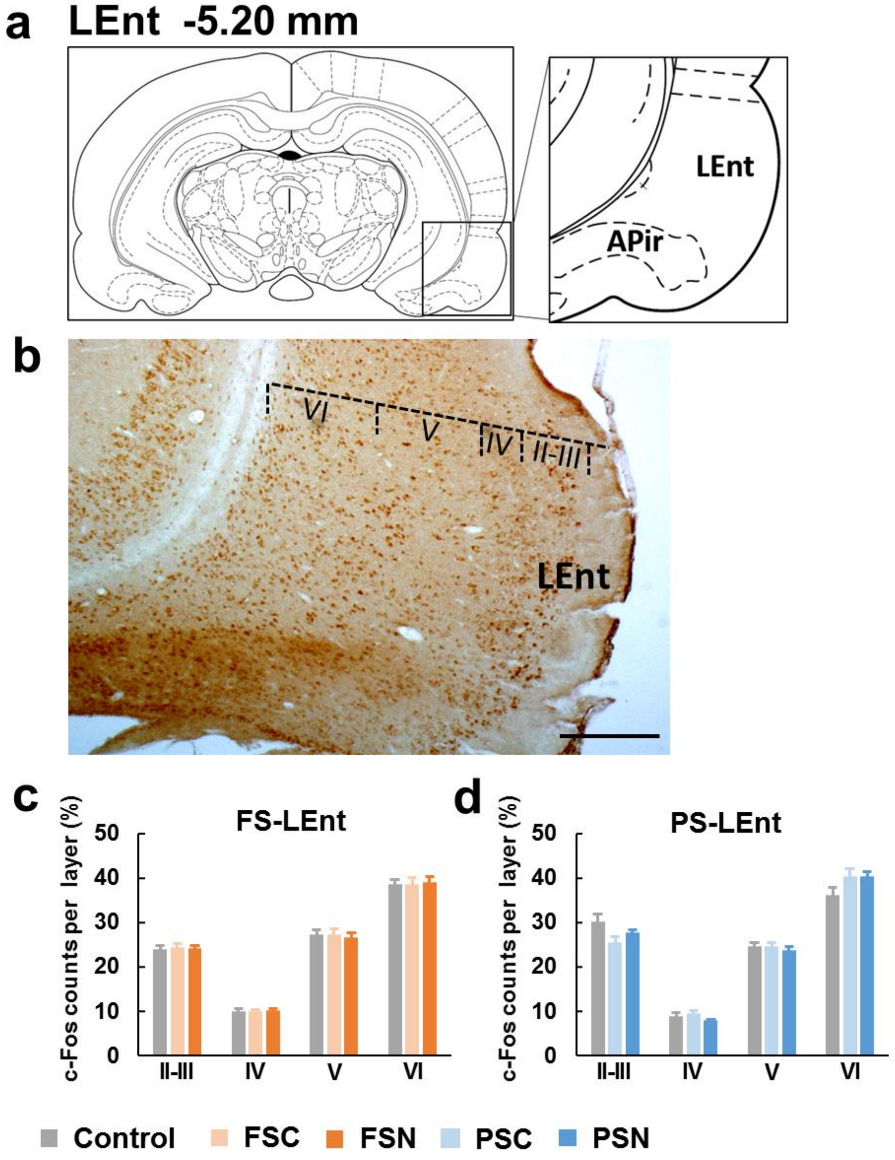

Figure S6: c-Fos expression in lateral entorhinal cortex. (**a**) Illustrations of brain sections of LEnt. (**b**) Photomicrographs of c-Fos staining in LEnt cortical layers I-VI. Scale bars, 200 µm. (**c**, **d**) c-Fos protein analysis revealed no difference among groups in LEnt. Data are represented as mean ± SEM. n = 5-7 per group. LEnt, Lateral Entorhinal Cortex.

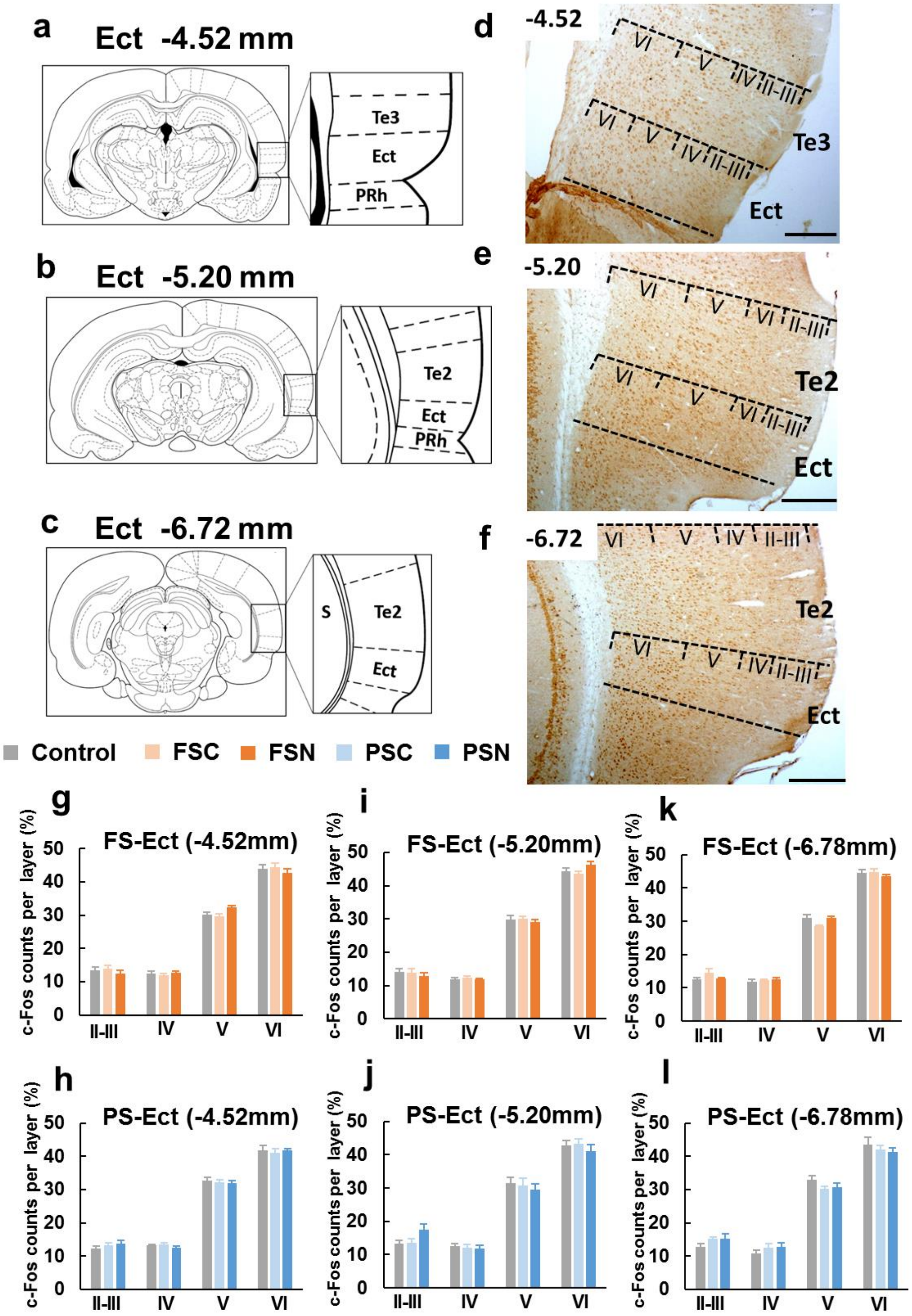

Figure S7. c-Fos expression in ectorhinal cortex (bregma -4.52, -5.20 and -6.72 mm). (**a**-**c**) Illustrations of brain sections of Ect (-4.52, -5.20 and -6.72 mm). (**d**-**f**) Photomicrographs of c-Fos staining in Ect (-4.52, -5.20 and -6.72 mm) cortical layers I-VI. Scale bars = 200 μm. (**g**, **h**) c-Fos protein analysis revealed no difference among groups in Ect (-4.52 mm). (**i**, **j**) c-Fos protein analysis revealed no difference among groups in Ect (-5.20 mm). (**k**, **l**) c-Fos protein analysis revealed no difference among groups in Ect (-6.72 mm). Data are represented as mean ± SEM. *n* = 6-7 per group. Ect, Ectorhinal Cortex.

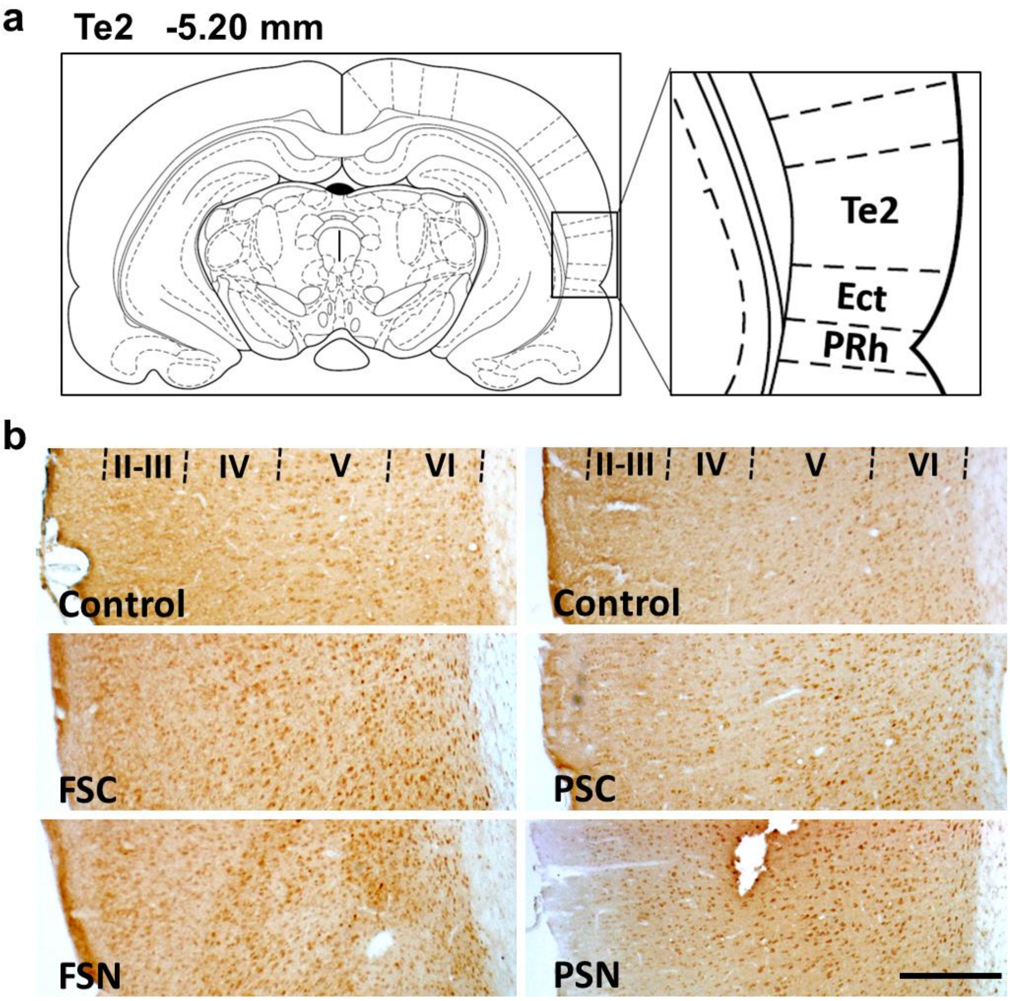

Figure S8. c-Fos distribution in PSN traumatic memory storage neocortex regions. (**a**) Illustrations of Te2 brain sections (bregma, -5.20 mm). (**b**) Photomicrographs of c-Fos staining within Te2 (-5.20 mm) cortical layers I-VI. Compared to control and PSC group, c-Fos counts of layers II-III significantly increased in PSN group. Scale bars = 200 μm.

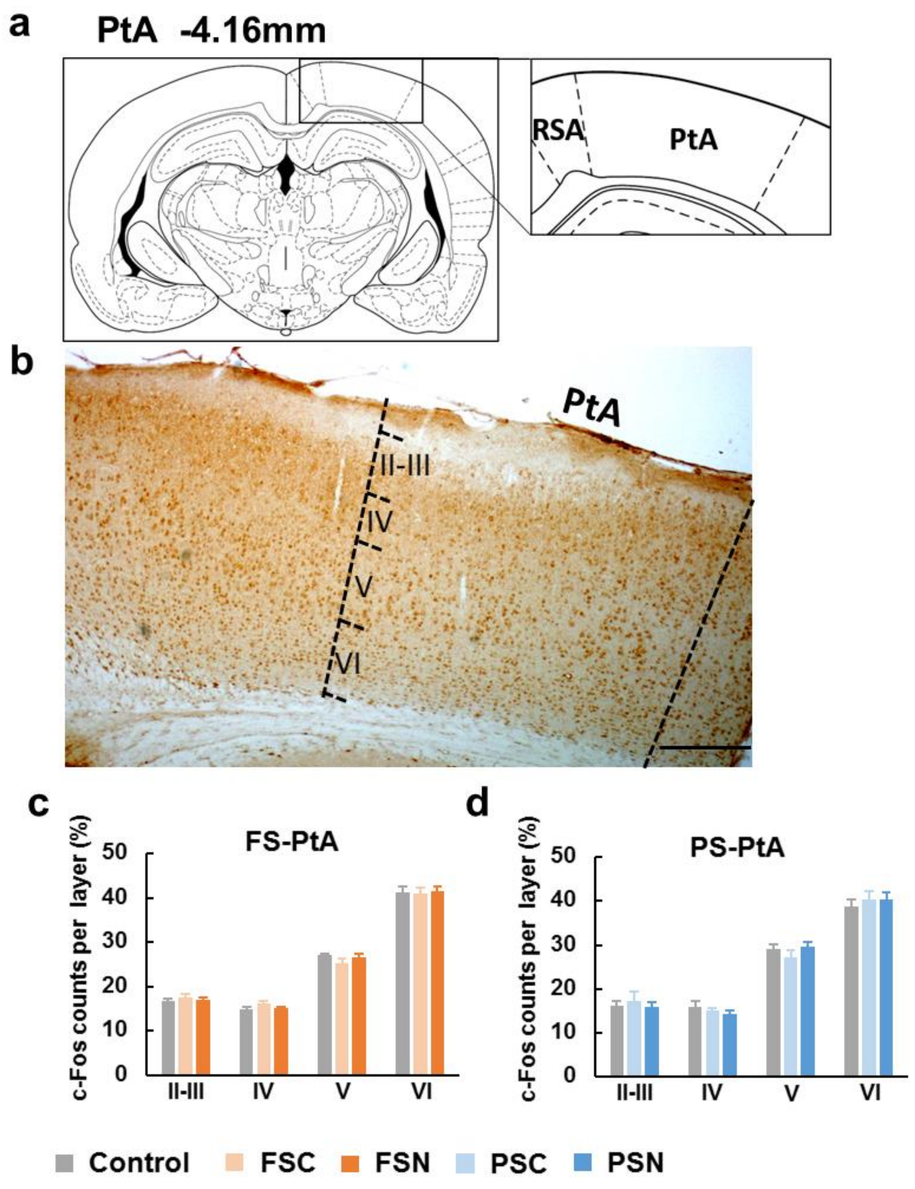

Figure S9. c-Fos expression in parietal association cortex. (**a**) Illustrations of brain sections of PtA. (**b**) Photomicrographs of c-Fos staining within PtA I-VI layers. Scale bars = 200 μm. (**c, d**) c-Fos protein analysis revealed no difference among groups in PtA. Data are represented as mean ± SEM. *n* = 6-7 per group. PtA, parietal association cortex.

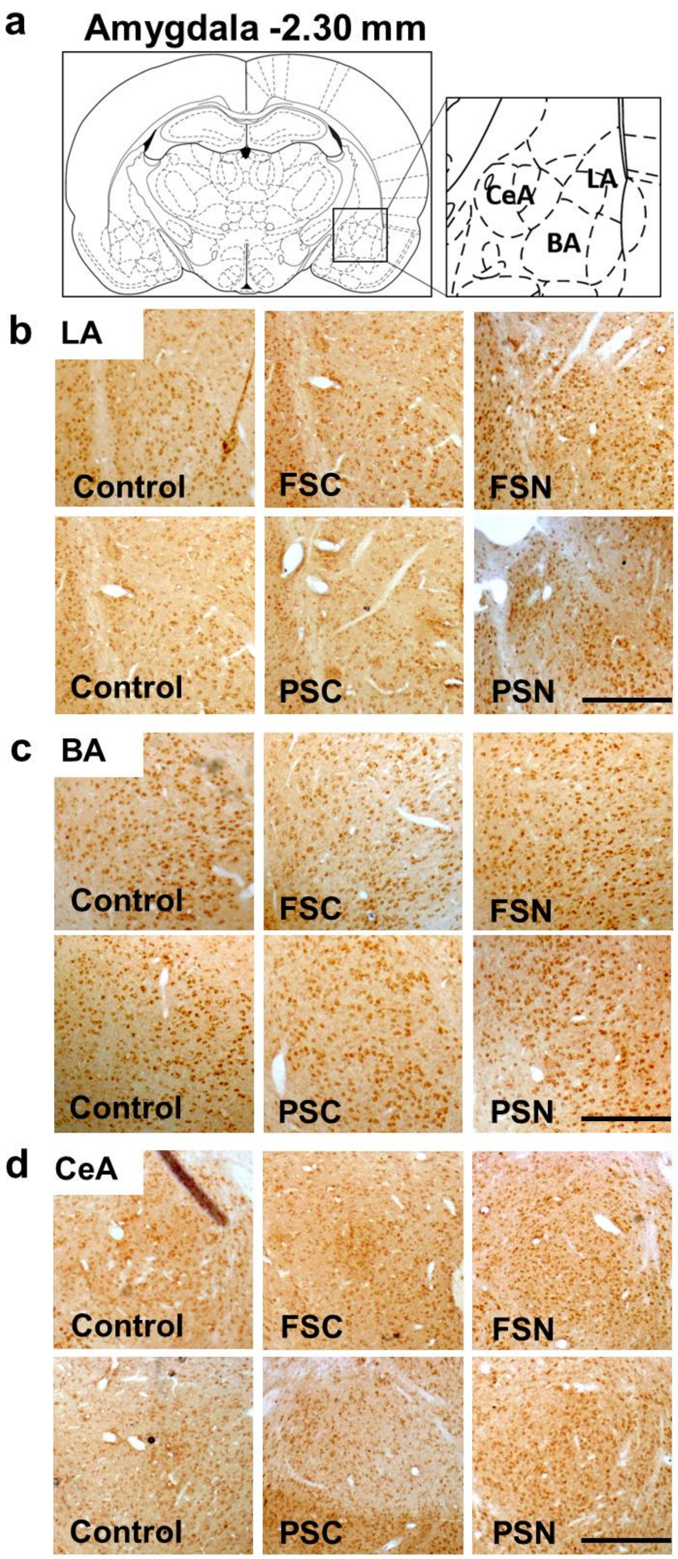

Figure S10. c-Fos expression in amygdala. (**a**) Illustrations of amygdala (LA, BA and CeA) brain sections. (**b**) Photomicrograph illustrating c-Fos expression in the LA. Fos expression in the LA significantly increased in FSN and PSN rats. (**c**) Photomicrograph illustrating c-Fos expression in the CeA. c-Fos expression in the CeA significantly increased in FSN and PSN rats. (**d**) Photomicrograph illustrating Fos expression in BA. Scale bars = 200 μm.

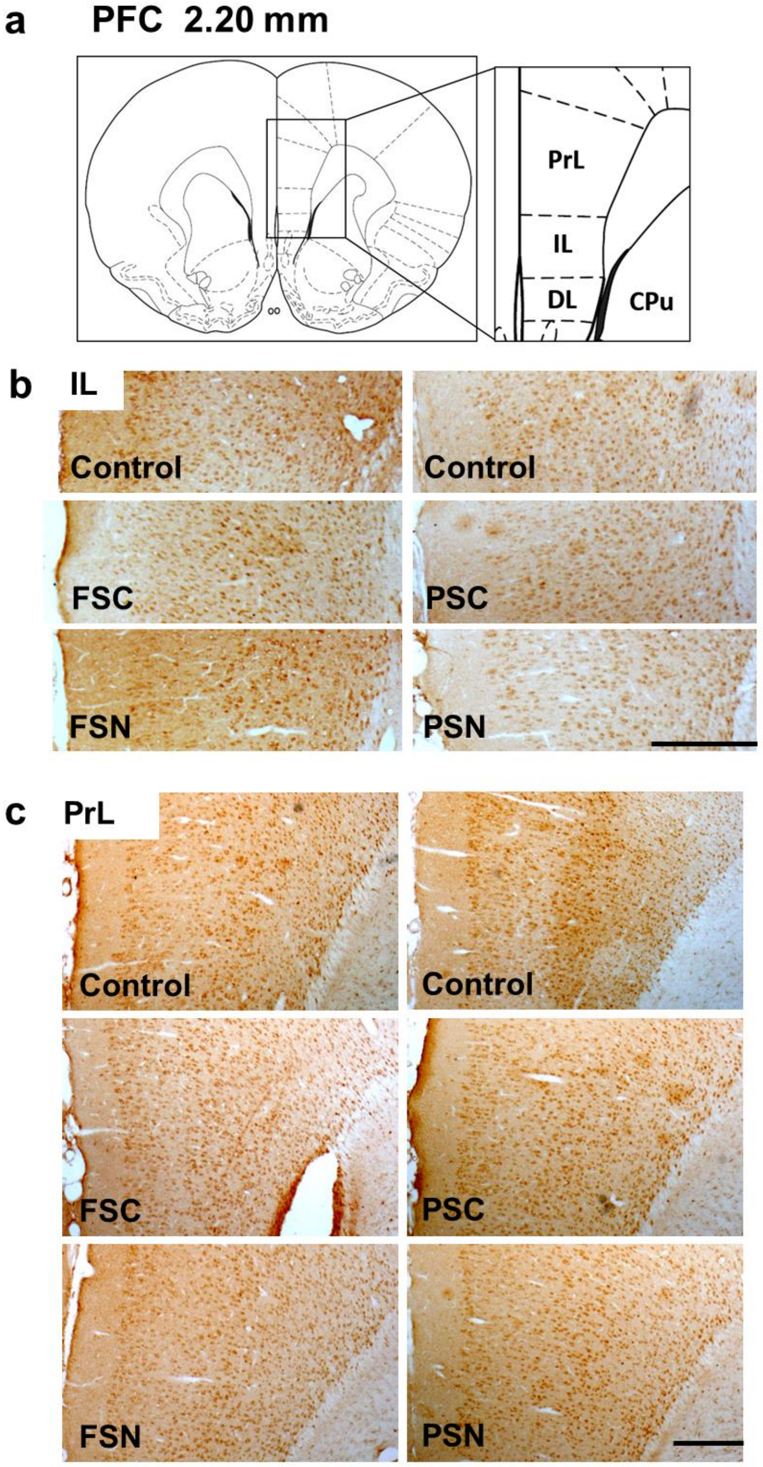

Figure S11. c-Fos expression in prefrontal cortex. (**a**) Illustrations of prefrontal cortex (IL and PrL) brain sections. (**b**) Photomicrograph illustrating c-Fos expression in the IL. c-Fos expression in the IL significantly decreased in both FSN and PSN rats. (**c**) Photomicrograph illustrating c-Fos expression in the PrL. Scale bars = 200 μm.

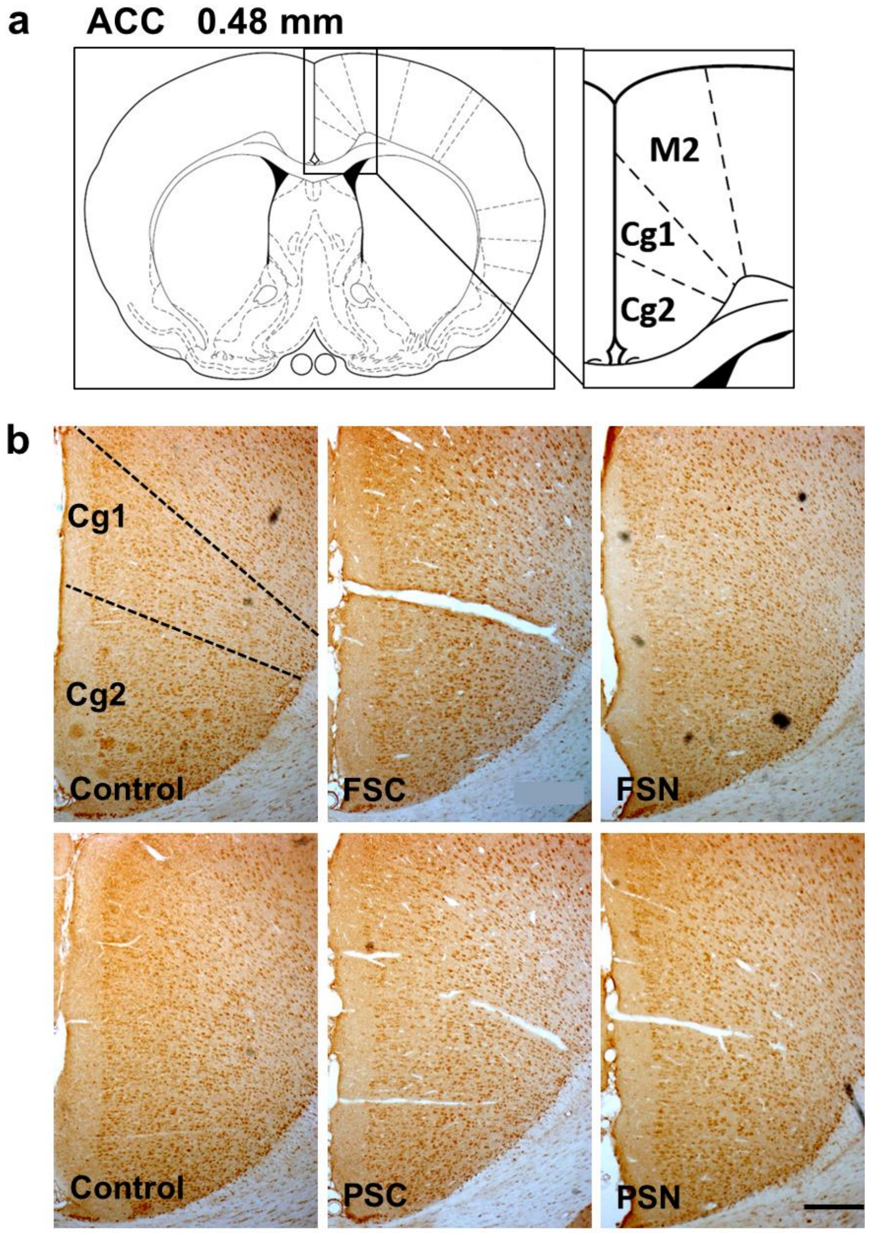

Figure S12. c-Fos expression in anterior cingulate cortex. (**a**) Illustrations of anterior cingulate cortex (Cg1 and Cg2) brain sections. (**b**) Photomicrograph illustrating c-Fos expression in the Cg1 and Cg2. c-Fos expression decreased in the Cg2 in both FSN and PSN groups, while the Cg1 activity was not changed. Scale bars = 200 μm.

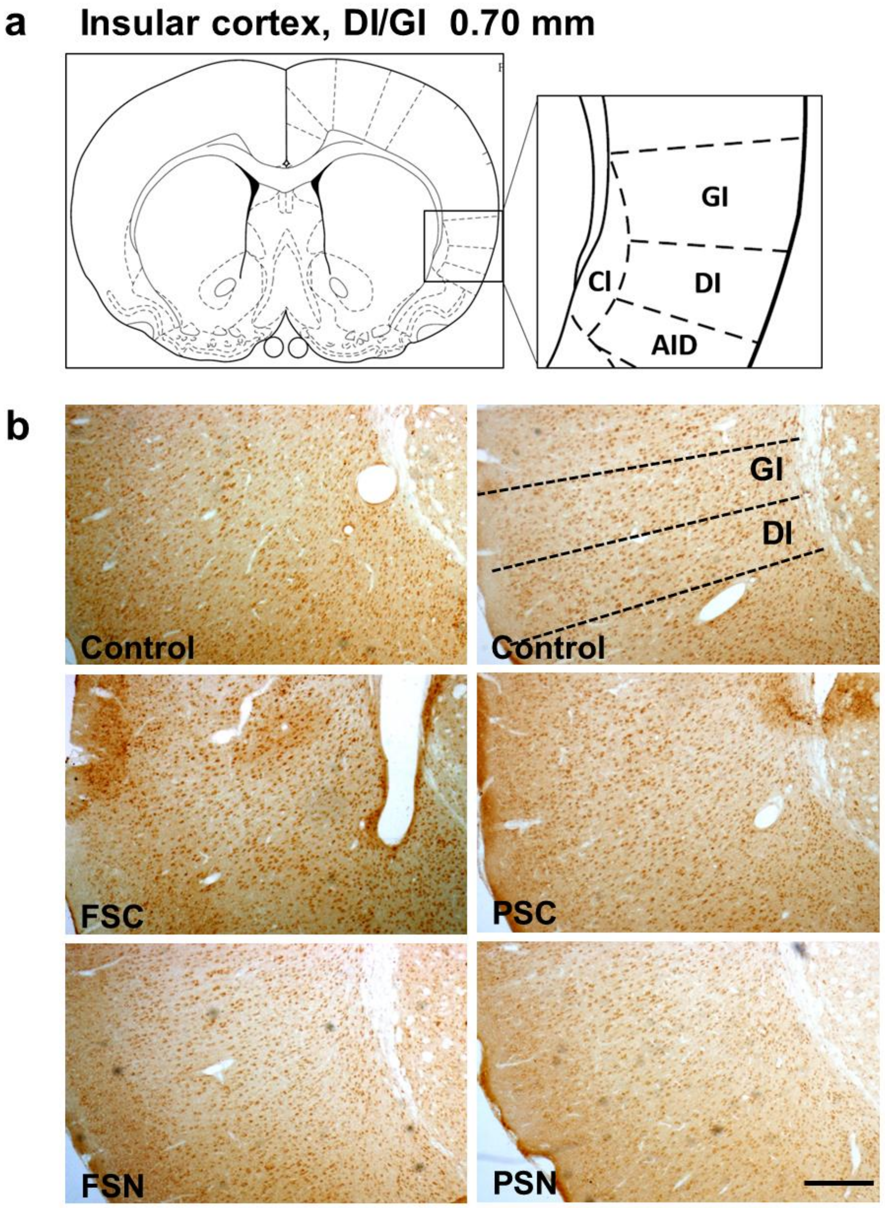

Figure S13. c-Fos expression in granular and dysgranular insular cortex. (**a**) Illustrations of the DI and GI brain sections. (**b**) Photomicrograph illustrating c-Fos expression in the DI and GI. The c-Fos expression in the DI and GI decreased in PSN group. Scale bars = 200 μm.

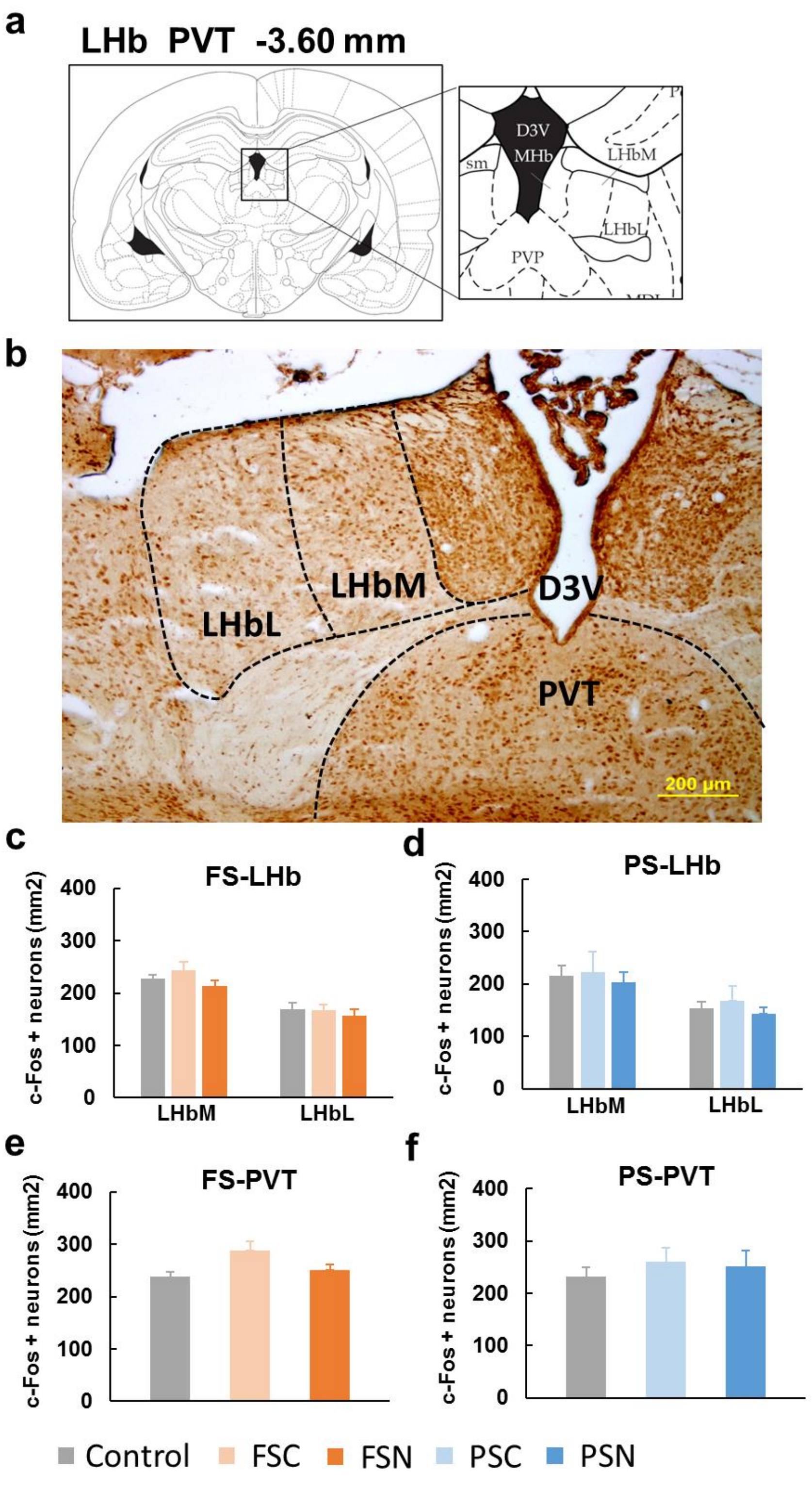

Figure S14. c-Fos expression in lateral habenular (LHbL and LHbM) and paraventricular thalamus (PVT). (**a**) Illustrations of the LHb and PVT brain sections. (**b**) Photomicrograph illustrating c-Fos expression in the LHb and PVT. Scale bars = 200 μm. (**c, d**) c-Fos protein analysis revealed no difference in the LHbL and LHbM among groups. (**e**, **f**) c-Fos protein expression was not different among groups in the PVT. Data are represented as mean ± SEM. *n* = 5-7.

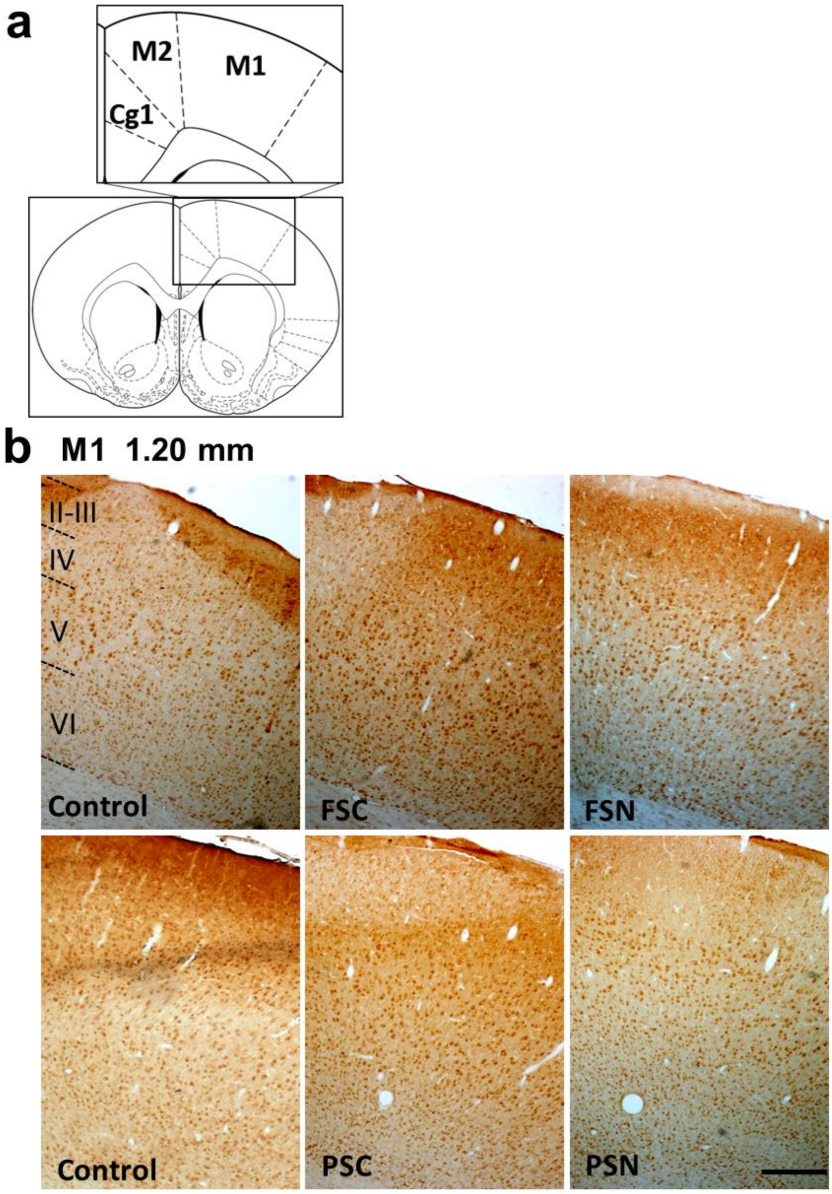

Figure S15. c-Fos expression in primary and secondary motor cortex. (**a**) Illustrations of the M1 and M2 brain sections. (**b**) Photomicrographs of c-Fos staining in M1 cortical layers I-VI, c-Fos expression in II-III layers significantly decreased in both FSN and PSN rats. Scale bars = 200 μm.

a

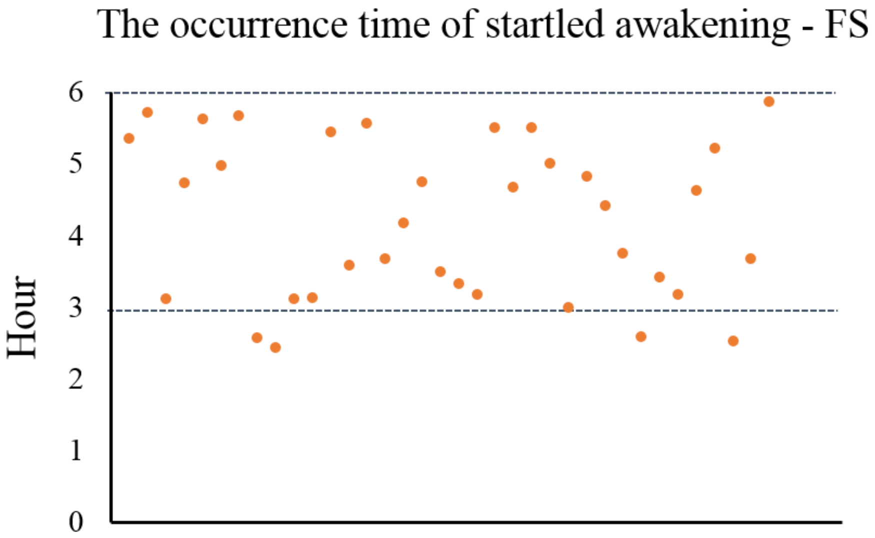

b

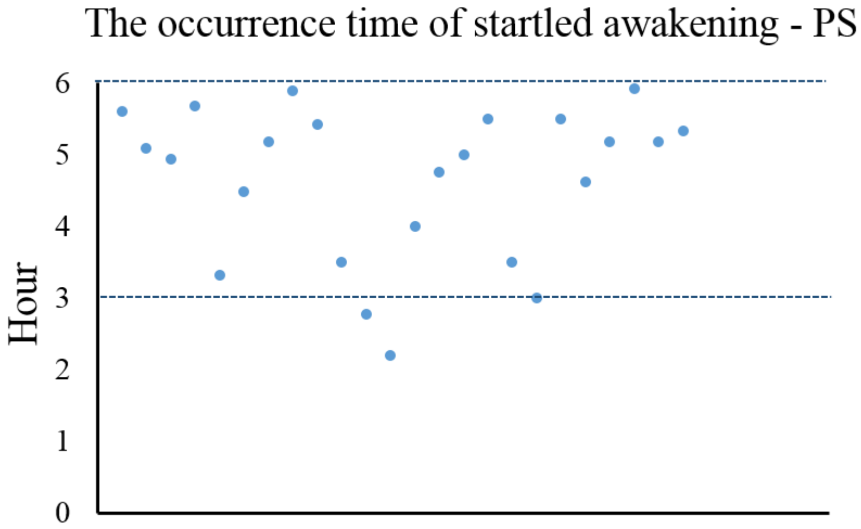

Figure S16. The majority of startled awakening events happened during 3-6 hour from the start of sleep recording. a for FSN, b for PSN.

Tables S1 to S7

Table S1. The incidence of startled awakening 21 days after stress exposure in different estrus phases

Group \ Estrus phase	Proestrus	Estrus	Metestrus	Diestrus
FSN	25.0%	16.6%	6.3%	8.3%
PSN	15.0%	12.5%	10.0%	9.4%

Table S2: Coronal sets of possible trauma-related memory storage cortex and fear emotional brain regions

**Brain regions of interest**	**Coronal to bregma (mm)**
Primary Somatosensory Cortex, jaw region (SIJ)	+2.70 ~ +2.20
Primary Somatosensory Cortex, hindlimb (S1HL)	-0.92 ~ -1.40
Primary Somatosensory Cortex, forelimb (S1FL)	-0.92 ~ -1.40
Secondary Somatosensory Cortex (S2)	-0.92 ~ -1.30
Primary Auditory Cortex (Au)	-5.20 ~ -5.60
Secondary visual cortex (V2MM)	-5.20 ~ -5.60
Secondary visual cortex (V2ML)	-5.20 ~ -5.60
Secondary visual cortex (V2L)	-5.20 ~ -5.60
Primary Visual Cortex, monocular area (V1M)	-6.72 ~ -7.04
Primary Visual Cortex, binocular area (V1B)	-6.72 ~ -7.04
Lateral Entorhinal Cortex (LEnt)	-5.20 ~ -5.60
Ectorhinal Cortex (Ect)	-4.16 ~ -4.52
Ectorhinal Cortex (Ect)	-5.20 ~ -5.60
Ectorhinal Cortex (Ect)	-6.72 ~ -7.04
Parietal Association Cortex (PtA)	-4.16 ~ -4.52
Temporal Association Cortex 3 (Te3)	-4.16 ~ -4.52
Temporal Association Cortex 2 (Te2)	-5.20 ~ -5.60

Temporal Association Cortex 2 (Te2)	-6.72 ~ -7.04
Primary Motor cortex (M1)	+1.20 ~ +0.70
Secondary Motor cortex (M2)	+1.20 ~ +0.70
Agranular Insular Cortex, ventral part (AIV)	+2.70 ~ +2.20
Agranular Insular Cortex, dorsal part (AID)	+2.70 ~ +2.20
Dysgranular Insular Cortex (DI)	+1.00 ~ +0.70
Granular insular cortex (GI)	+1.00 ~ +0.70
Amygdala, lateral part (LA)	-2.30 ~ -2.56
Amygdala, basal part (BA)	-2.30 ~ -2.56
Amygdala, central part (CeA)	-2.30 ~ -2.56
Prefrontal cortex, infralimbic cortex (IL)	+2.70 ~ +2.20
Prefrontal cortex, prelimbic cortex (PrL)	+2.70 ~ +2.20
Anterior cingulate cortex, dorsal part (Cg1)	+0.70 ~ +0.20
Anterior cingulate cortex, caudal part (Cg2)	+0.70 ~ +0.20
Lateral habenular nucleus, lateral part (LHbL)	-3.30 ~ -3.80
Lateral habenular nucleus, medial part (LHbM)	-3.30 ~ -3.80
Paraventricular thalamic nucleus (PVT)	-3.30 ~ -3.80

Table S3. Summary of statistical results. Fos distribution in II-VI layers in the interested neocortex.

Brain region	group	II-III	IV	V	VI
Primary Somatosensory Cortex, jaw region (SIJ)	FS	$F_{2,17} = 3.126$ $p = 0.070$	$F_{2,17} = 0.415$ $p = 0.667$	$F_{2,17} = 0.756$ $p = 0.485$	$F_{2,17} = 0.475$ $p = 0.630$
	PS	$F_{2,15} = 1.810$ $p = 0.198$	$F_{2,15} = 0.938$ $p = 0.413$	$F_{2,15} = 0.820$ $p = 0.459$	$F_{2,15} = 2.400$ $p = 0.125$
Primary Somatosensory Cortex, hindlimb (S1HL)	FS	$F_{2,17} = 0.432$ $p = 0.656$	$F_{2,17} = 0.227$ $p = 0.800$	$F_{2,17} = 0.646$ $p = 0.536$	$F_{2,17} = 2.354$ $p = 0.125$
	PS	$F_{2,15} = 0.197$ $p = 0.823$	$F_{2,15} = 0.575$ $p = 0.575$	$F_{2,15} = 0.129$ $p = 0.880$	$F_{2,15} = 1.389$ $p = 0.280$
Primary Somatosensory Cortex, forelimb (S1FL)	FS	$F_{2,17} = 0.207$ $p = 0.815$	$F_{2,17} = 0.407$ $p = 0.672$	$F_{2,17} = 1.989$ $p = 0.167$	$F_{2,17} = 1.000$ $p = 0.388$
	PS	$F_{2,15} = 0.852$ $p = 0.446$	$F_{2,15} = 2.805$ $p = 0.092$	$F_{2,15} = 1.819$ $p = 0.196$	$F_{2,15} = 1.455$ $p = 0.265$
Secondary Somatosensory Cortex (S2)	FS	$F_{2,15} = 8.210$ $p = 0.004$	$F_{2,15} = 1.816$ $p = 0.197$	$F_{2,15} = 5.177$ $p = 0.020$	$F_{2,15} = 0.855$ $p = 0.445$
	PS	$F_{2,15} = 0.028$ $p = 0.973$	$F_{2,15} = 0.294$ $p = 0.750$	$F_{2,15} = 0.181$ $p = 0.837$	$F_{2,15} = 0.087$ $p = 0.917$
Primary Auditory Cortex (Au)	FS	$F_{2,51} = 12.60$ $p < 0.001$	$F_{2,51} = 0.798$ $p = 0.456$	$F_{2,51} = 1.791$ $p = 0.177$	$F_{2,51} = 4.456$ $p = 0.016$
	PS	$F_{2,15} = 1.524$ $p = 0.250$	$F_{2,15} = 0.324$ $p = 0.728$	$F_{2,15} = 0.701$ $p = 0.512$	$F_{2,15} = 0.707$ $p = 0.509$
Secondary visual cortex (V2MM)	FS	$F_{2,13} = 1.068$, $p = 0.372$;	$F_{2,13} = 1.962$ $p = 0.180$	$F_{2,13} = 1.096$ $p = 0.363$	$F_{2,13} = 0.774$ $p = 0.481$
	PS	$F_{2,14} = 2.127$ $p = 0.156$	$F_{2,14} = 0.628$ $p = 0.548$	$F_{2,14} = 0.385$ $p = 0.687$	$F_{2,14} = 0.486$ $p = 0.625$
Secondary visual cortex (V2ML)	FS	$F_{2,14} = 0.957$ $p = 0.408$	$F_{2,14} = 1.195$ $p = 0.332$	$F_{2,14} = 3.662$ $p = 0.053$	$F_{2,14} = 0.589$ $p = 0.568$
	PS	$F_{2,14} = 0.267$ $p = 0.769$	$F_{2,14} = 0.027$ $p = 0.973$	$F_{2,14} = 0.563$ $p=0.582$	$F_{2,14} = 0.027$ $p = 0.973$
Secondary visual cortex (V2L)	FS	$F_{2,13} = 0.163$ $p = 0.851$	$F_{2,13} = 0.882$ $p = 0.437$	$F_{2,13} = 0.046$ $p = 0.955$	$F_{2,13} = 0.566$ $p = 0.581$
	PS	$F_{2,14} = 0.698$ $p = 0.513$	$F_{2,14} = 0.195$ $p = 0.825$	$F_{2,14} = 1.095$ $p = 0.360$	$F_{2,14} = 0.671$ $p = 0.526$
Primary Visual Cortex, monocular area (V1M)	FS	$F_{2,20} = 0.153$ $p = 0.859$	$F_{2,20} = 0.128$ $p = 0.880$	$F_{2,20} = 0.483$ $p = 0.624$	$F_{2,20} = 0.222$ $p = 0.803$
	PS	$F_{2,15} = 1.799$ $p = 0.199$	$F_{2,15} = 0.056$ $p = 0.946$	$F_{2,15} = 0.353$ $p = 0.708$	$F_{2,15} = 0.600$ $p = 0.561$

Primary Visual Cortex, binocular area (V1B)	FS	$F_{2,20} = 0.484$ $p = 0.624$	$F_{2,20} = 0.548$ $p = 0.587$	$F_{2,20} = 3.259$ $p = 0.060$	$F_{2,20} = 0.222$ $p = 0.803$
	PS	$F_{2,15} = 2.217$ $p = 0.143$	$F_{2,15} = 0.693$ $p = 0.516$	$F_{2,15} = 0.101$ $p = 0.905$	$F_{2,15} = 0.547$ $p = 0.590$
Lateral Entorhinal Cortex (LEnt)	FS	$F_{2,17} = 0.173$ $p = 0.843$	$F_{2,17} = 0.016$ $p = 0.984$	$F_{2,17} = 0.055$ $p = 0.947$	$F_{2,17} = 0.163$ $p = 0.851$
	PS	$F_{2,12} = 3.632$ $p = 0.058$	$F_{2,12} = 1.626$ $p = 0.237$	$F_{2,12} = 0.437$ $p = 0.656$	$F_{2,12} = 2.572$ $p = 0.118$
Ectorhinal Cortex (Ect) -4.52mm	FS	$F_{2,18} = 0.827$ $p = 0.453$	$F_{2,18} = 0.652$ $p = 0.533$	$F_{2,18} = 3.385$ $p = 0.056$	$F_{2,18} = 0.582$ $p = 0.569$
	PS	$F_{2,15} = 1.013$ $p = 0.387$	$F_{2,15} = 0.832$ $p = 0.454$	$F_{2,15} = 0.148$ $p = 0.864$	$F_{2,15} = 0.148$ $p = 0.864$
Ectorhinal Cortex (Ect) -5.20mm	FS	$F_{2,18} = 0.375$ $p = 0.692$	$F_{2,18} = 0.829$ $p = 0.452$	$F_{2,18} = 0.362$ $p = 0.701$	$F_{2,18} = 2.087$ $p = 0.153$
	PS	$F_{2,15} = 2.609$ $p = 0.107$	$F_{2,15} = 0.117$ $p = 0.891$	$F_{2,15} = 0.283$ $p = 0.758$	$F_{2,15} = 0.480$ $p = 0.628$
Ectorhinal Cortex (Ect) -6.72mm	FS	$F_{2,18} = 1.638$ $p = 0.222$	$F_{2,18} = 0.665$ $p = 0.527$	$F_{2,18} = 3.334$ $p = 0.059$	$F_{2,18} = 0.619$ $p = 0.549$
	PS	$F_{2,15} = 1.539$ $p = 0.247$	$F_{2,15} = 1.250$ $p = 0.315$	$F_{2,15} = 1.601$ $p = 0.234$	$F_{2,15} = 0.393$ $p = 0.682$
Parietal Association Cortex (PtA)	FS	$F_{2,17} = 0.620$ $p = 0.550$	$F_{2,17} = 2.576$ $p = 0.105$	$F_{2,17} = 1.179$ $p = 0.331$	$F_{2,17} = 0.044$ $p = 0.957$
	PS	$F_{2,15} = 0.198$ $p = 0.822$	$F_{2,15} = 0.972$ $p = 0.401$	$F_{2,15} = 0.801$ $p = 0.467$	$F_{2,15} = 0.315$ $p = 0.735$
Temporal Association Cortex (Te3) -4.52mm	FS	$F_{2,18} = 0.244$ $p = 0.786$	$F_{2,18} = 3.479$ $p = 0.053$	$F_{2,18} = 1.325$ $p = 0.290$	$F_{2,18} = 0.073$ $p = 0.930$
	PS	$F_{2,15} = 3.434$ $p = 0.059$	$F_{2,15} = 0.260$ $p = 0.774$	$F_{2,15} = 2.280$ $p = 0.137$	$F_{2,15} = 0.106$ $p = 0.901$
Temporal Association Cortex (Te2) -5.20mm	FS	$F_{2,18} = 0.398$ $p = 0.677$	$F_{2,18} = 0.293$ $p = 0.749$	$F_{2,18} = 0.059$ $p = 0.943$	$F_{2,18} = 0.189$ $p = 0.829$
	PS	$F_{2,15} = 5.782$ $p = 0.014$	$F_{2,15} = 0.194$ $p = 0.826$	$F_{2,15} = 0.157$ $p = 0.856$	$F_{2,15} = 2.978$ $p = 0.081$
Temporal Association Cortex (Te2) -6.72mm	FS	$F_{2,18} = 2.408$ $p = 0.118$	$F_{2,18} = 0.467$ $p = 0.635$	$F_{2,18} = 1.808$ $p = 0.192$	$F_{2,18} = 0.649$ $p = 0.535$
	PS	$F_{2,15} = 1.489$ $p = 0.257$	$F_{2,15} = 0.071$ $p = 0.932$	$F_{2,15} = 1.860$ $p = 0.190$	$F_{2,15} = 1.802$ $p = 0.199$
Primary Motor cortex (M1)	FS	$F_{2,18} = 4.637$ $p =0.024$	$F_{2,18} = 0.002$ $p =0.988$	$F_{2,18} = 3.024$ $p =0.074$	$F_{2,18} = 0.621$ $p = 0.549$
	PS	$F_{2,21} = 19.34$ $p < 0.001$	$F_{2,21} = 1.013$ $p = 0.380$	$F_{2,21} = 2.960$ $p = 0.080$	$F_{2,21} = 1.682$ $p = 0.210$

Secondary Motor cortex (M2)	FS	$F_{2,17}$ = 0.626 p = 0.546	$F_{2,17}$ = 0.145, p = 0.866	$F_{2,17}$ = 1.607 p = 0.230	$F_{2,17}$ = 1.163 p = 0.336
	PS	$F_{2,21}$ = 0.643 p = 0.536	$F_{2,21}$ = 0.055 p = 0.947	$F_{2,21}$ = 0.160 p = 0.853	$F_{2,21}$ = 0.229 p = 0.798

Table S4. Summary of statistical results. Fos expression in emotional regulation brain region.

Brain region	group	F&P value
Agranular Insular Cortex, ventral part (AIV)	FS	$F_{2,17} = 0.104$, $p = 0.902$
	PS	$F_{2,17} = 0.115$, $p = 0.892$
Agranular Insular Cortex, dorsal part (AID)	FS	$F_{2,17} = 1.133$, $p = 0.345$
	PS	$F_{2,17} = 1.293$, $p = 0.300$
Dysgranular Insular Cortex (DI)	FS	$F_{2,17} = 0.781$, $p = 0.474$
	PS	$F_{2,18} = 5.594$, $p = 0.013$
Granular insular cortex (GI)	FS	$F_{2,17} = 0.869$, $p = 0.437$
	PS	$F_{2,18} = 4.339$, $p = 0.029$
Amygdala, lateral part (LA)	FS	$F_{2,27} = 28.452$, $p < 0.001$
	PS	$F_{2,21} = 8.056$, $p = 0.003$
Amygdala, basal part (BA)	FS	$F_{2,27} = 1.800$, $p = 0.185$
	PS	$F_{2,21} = 2.523$, $p = 0.104$
Amygdala, central part (CeA)	FS	$F_{2,27} = 17.517$, $p < 0.001$
	PS	$F_{2,21} = 5.154$, $p = 0.015$
Prefrontal cortex, infralimbic cortex (IL)	FS	$F_{2,27} = 3.905$, $p = 0.032$
	PS	$F_{2,18} = 5.027$, $p = 0.013$
Prefrontal cortex, prelimbic cortex (PrL)	FS	$F_{2,27} = 0.078$, $p = 0.925$
	PS	$F_{2,18} = 0.654$, $p = 0.532$
Anterior cingulate cortex, dorsal part (CG1)	FS	$F_{2,25} = 0.868$, $p = 0.432$
	PS	$F_{2,24} = 1.302$, $p = 0.291$
Anterior cingulate cortex, caudal part (CG2)	FS	$F_{2,25} = 3.897$, $p = 0.034$
	PS	$F_{2,24} = 4.161$, $p = 0.028$
Lateral habenular Nucleus, medial part (LHbM)	FS	$F_{2,18} = 0.959$, $p = 0.403$
	PS	$F_{2,12} = 0.124$, $p = 0.885$
Lateral habenular Nucleus, lateral part (LHbL)	FS	$F_{2,18} = 0.015$, $p = 0.985$
	PS	$F_{2,12} = 0.453$, $p = 0.646$
Paraventricular thalamic nucleus (PVT)	FS	$F_{2,18} = 1.414$, $p = 0.270$
	PS	$F_{2,18} = 0.300$, $p = 0.744$

Table S5. Control of FS vs. control of PS – EEG band

EEG band	Figure	Statistic
**Delta**	1	*t* = 0.825,df = 22, *p* = 0.418
**Theta**	1	*t* = 0.914,df = 22, *p* = 0.371
**Beta**	1	*t* = -1.137,df = 22, *p* = 0.268
**Alpha**	1	*t* = -0.523,df = 22, *p* = 0.606

Table S6. Control of FS vs. control of PS – interested cortical regions

Cortical regions	Figure	II-III	IV	V	VI
**Au**	2	*t*=-1.499,df=26, *p*=0.146	*t*=1.541,df=26, *p*=0.135	*t*=0.239,df=26, *p*=0.813	*t*=-0.615,df=26, *p*=0.544
**S2**	2	*t*=-1.217,df=10, *p*=0.251	*t*=0.291,df=10, *p*=0.777	*t*=0.831,df=10, *p*=0.425	*t*=1.781,df=10, *p*=0.105
**Te3(-4.52 mm)**	3	*t*=0.649,df=11, *p*=0.530	*t*=-1.246,df=11, *p*=0.239	*t*=-1.849,df=11, *p*=0.091	*t*=1.187,df=11, *p*=0.260
**Te2(-5.20 mm)**	3	*t*=0.717,df=11, *p*=0.488	*t*=-0.749,df=11, *p*=0.470	*t*=-0.567,df=11, *p*=0.582	*t*=0.453,df=11, *p*=0.659
**Te2(-6.72 mm)**	3	*t*=-0.363,df=11, *p*=0.723	*t*=-0.789,df=11, *p*=0.447	*t*=0.048,df=11, *p*=0.963	*t*=0.641,df=11, =0.535
**M1**	5	*t*=0.516,df=13, *p*=0.614	*t*=0.628,df=13, *p*=0.541	*t*=0.090,df=13, *p*=0.929	*t*=-0.949,df=13, *p*=0.360
**M2**	5	*t*=2.215,d=13, *p*=0.045	*t*=1.184,df=13, *p* =0.258	*t*=-1.492,df=13, *p*=0.160	*t*=-1.245,df=13, *p*=0.235
**S1FL**	S3	*t*=0.425,df=11, *p*=0.679	*t*=-0.538,df=11, *p*=0.601	*t*=0.166,df=11, *p*=0.872	*t*=-0.017,df=11, *p*=0.987
**S1HL**	S3	*t*=1.215,df=11, *p*=0.250	*t*=1.885,df=11, *p*=0.086	*t*=-0.982,df=11, *p*=0.347	*t*=-1.390,df=11, *p*=0.192
**S1J**	S3	*t*=0.935,df=11, *p*=0.370	*t*=1.292,df=11, *p*=0.223	*t*=-0.235,df=11, *p*=0.818	*t*=-1.399,df=11, *p*=0.189
**V1M**	S4	*t*=-1.684,df=12, *p*=0.118	*t*=-0.064,df=12, *p*=0.950	*t*=0.330,df=12, *p*=0.747	*t*=0.767,df=12, *p*=0.458
**V1B**	S4	*t*=-1.861,df=12, *p*=0.087	*t*=0.552,df=12, *p*=0.591	*t*=2.089,df=12, *p*=0.059	*t*=-1.146,df=12, *p*=0.274
**V2MM**	S5	*t*=-1.651,df=10, *p*=0.130	*t*=-1.259,df=10, *p*=0.237	*t*=2.023,df=10, *p*=0.071	*t*=1.434,df=10, *p*=0.182
**V2ML**	S5	*t*=-0.555,df=11, *p*=0.590	*t*=-1.761,df=11, *p*=0.106	*t*=0.979,df=11, *p*=0.349	*t*=0.650,df=11, *p*=0.529

**V2L**	S5	*t*=0.778,df=10, *p*=0.455	*t*=1.039,df=10, *p*=0.323	*t*=1.215,df=10, *p*=0.252	*t*=-0.679,df=10, *p*=0.513
**LEnT**	S6	*t*=-1.897,df=10, *p*=-0.087	*t*=1.153,df=10, *p*=0.276	*t*=0.516,df=10, *p*=0.617	*t*=0.830,df=10, *p*=0.426
**Ect(-4.52 mm)**	S7	*t*=1.045,df=11, *p*=0.318	*t*=-0.931,df=11, *p*=0.372	*t*=-1.988,df=11, *p*=0.072	*t*=0.981,df=11, *p*=0.348
**Ect(-5.20 mm)**	S7	*t*=0.471,df=11, *p*=0.647	*t*=-0.796,df=11, *p*=0.443	*t*=-0.798,df=11, *p*=0.442	*t*=0.846,df=11, *p*=0.416
**Ect(-6.72 mm)**	S7	*t*=-0.385,df=11, *p* =0.708	*t*=0.944,df=11, *p*=0.366	*t*=-1.204,df=11, *p*=0.254	*t*=0.489,df=11, *p*=0.635
**PtA**	S9	*t*=0.495,df=11, *p*=0.630	*t*=-0.940,df=11, *p*=0.367	*t*=-1.477,df=11, *p*=0.176	*t*=1.376,df=11, *p*=0.196

Table S7. Control of FS vs. control of PS – emotional regulation brain regions

**Brain regions**	**Figure**	**Statistics**
**Amygdala-LA**	4	*t* = 0.336,df = 16, *p* = 0.741
**Amygdala-BA**	4	*t* = 0.972,df = 16, *p* = 0.345
**Amygdala-CeA**	4	*t* = -1.252,df = 16, *p* = 0.229
**PFC-IL**	4	*t* = -1.211,df = 15, *p* = 0.245
**PFC-PrL**	4	*t* = -1.133,df = 15, *p* = 0.275
**ACC-Cg1**	4	*t* = 0.219,df = 17, *p* = 0.830
**ACC-Cg2**	4	*t* = -0.088,df = 17, *p* = 0.931
**DI**	4	*t* = -1.613,df = 12, *p* = 0.133
**GI**	4	*t* = -1.235,df = 12, *p* = 0.241
**AIV**	4	*t* = -1.129,df = 12, *p* = 0.281
**AID**	4	*t* = -0.313,df = 12, *p* = 0.759
**LHb-LHbL**	S14	*t* = 0.603,df = 10, *p* = 0.560
**LHb-LHbM**	S14	*t* = 0.879,df = 10, *p* = 0.400
**PVT**	S14	*t* = 0.280,df = 12, *p* = 0.784

Captions for Videos S1 to S3

Video S1: Normal awakening.

Video S2: FSN rats came to startle awake.

Video S3: PSN rats came to startle awake.
